# Nonlinear Optical Response in Layer‐Stacked Gallenene with Ferroelectric Polarization

**DOI:** 10.1002/adma.202501058

**Published:** 2025-08-21

**Authors:** Muhammad Yunusa, Andrew K. Schulz, Tim Parker, Felix Schneider, Kenan Elibol, Marius Predel, Jana Dzíbelová, Michel Rebmann, Taylan Gorkan, Jiahao Ye, Jin‐Chong Tan, Wenbin Kang, Peter A. van Aken, Alfred J. Meixner, Engin Durgun, Jani Kotakoski, Dai Zhang, Metin Sitti

**Affiliations:** ^1^ Physical Intelligence Department Max Planck Institute for Intelligent Systems 70569 Stuttgart Germany; ^2^ Max Planck Institute for Intelligent Systems 70569 Stuttgart Germany; ^3^ Institute for Physical and Theoretical Chemistry University of Tübingen and LISA+ 72076 Tübingen Germany; ^4^ Max Planck Institute for Solid State Research 70569 Stuttgart Germany; ^5^ Faculty of Physics University of Vienna Boltzmanngasse 5 Vienna 1090 Austria; ^6^ UNAM‐National Nanotechnology Research Center and Institute of Materials Science and Nanotechnology Bilkent University Ankara 06800 Turkey; ^7^ Multifunctional Materials and Composites (MMC) Laboratory Department of Engineering Science University of Oxford Oxford OX1 3PJ UK; ^8^ Department of Mechanical Engineering City University of Hong Kong 83 Tat Chee Avenue Kowloon 99907 Hong Kong; ^9^ School of Medicine and College of Engineering Koç University Istanbul 34450 Turkey

**Keywords:** 2D gallenene, ferroelectricity, liquid metal, phase transition, second harmonic generation

## Abstract

Polar metals are very rare and challenging to realize due to the incompatibility of ferroelectricity and metallicity. Mobile electrons in polar metals effectively screen the static electric field and dipoles. Recent studies show that 2D van der Waals metals without an inversion center can have polar order due to specific layer stacking. However, room temperature reversible ferroelectricity and nonlinear second harmonic generation in non‐centrosymmetric polar metals remain unrealized. Here, the experimental realization of AB‐stacked gallenene (a100) nanocrystals with a room temperature ferroelectric polarization in a liquid gallium environment is reported. Using first‐principles calculations, the origin of spontaneous polarization (Ps) due to a broken symmetry in multilayer gallenene structures, resulting in P1 (space group) and C1 (point group) symmetry is explained. The reversible polarization switching is characterized using piezoresponse force microscopy. This results demonstrate the reversible nonlinear optical response of the AB‐stacked gallenene crystal through second harmonic generation (SHG) microscopy. The intensities of SHG signals are controlled via angular rotations and thermal heating, which indicate a phase transition at high temperatures. Furthermore, electrical perturbation enables the tunability of SHG intensity. Bipolar resistive switching is demonstrated in a two‐terminal device. These findings open avenues for advancements in 2D ferroelectricity, piezoelectricity, and topological superconductivity.

## Graphical abstract

Liquid metals are utilized in applications ranging from soft robotics to conductive inks, owing to their physical properties providing distinct mechanical, electrical, and thermal advantages. This work uses classical material science techniques to characterize ferroelectric polarization in supercooled liquid gallium and utilizes second harmonic generation imaging for further perturbation analysis.

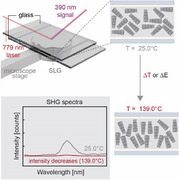

## 1 Introduction

Coexistence of ferroelectricity and metallicity is forbidden due to the effective electric field and dipole screening of electrons in metals. In 1965, Anderson and Blunt^[^ ^1^ ^]^ suggested a ferroelectric‐like martensitic transition in metals due to the appearance of a polar axis and broken inversion symmetry. Recently, there has been increasing interest in metallic ferroelectricity and unconventional 2D sliding ferroelectric materials. The first evidence of metallic and ferroelectric metal was reported in bulk crystalline van der Waals WTe_2_ in 2019.^[^ ^2^ ^]^ With a black phosphorus‐like configuration, 2D ferroelectricity in a single‐element bismuth monolayer has been demonstrated with in‐plane electric polarization.^[^ ^3^ ^]^ Moreover, 2D materials such as graphene,^[^ ^4^ ^]^ boron nitride,^[^ ^5^, ^6^ ^]^ indium selenide,^[^ ^7^ ^]^ and transition metal dichalcogenide heterostructures^[^ ^8^, ^9^ ^]^ are shown to reversibly switch their ferroelectric polarization in an applied electric field. Recently, air‐stable polar 2D metals of gallium and indium (Ga and In) with out‐of‐plane polarization have been realized in hermetic seals through confinement by graphene layers in a hetero‐epitaxy technique.^[^ ^10^, ^11^ ^]^ The environmentally stable 2D polar metals (Ga and In) have demonstrated intriguing nonlinear optical properties, including Raman response and extraordinary second harmonic generation with the largest second‐order susceptibilities ever reported in two‐to‐three atom‐thick Ga and In films.^[^ ^10^, ^11^, ^12^, ^13^ ^]^

By the solid‐melt exfoliation technique, Kochat et al.^[^ ^14^ ^]^ have discovered 2D gallium crystals (aka gallenene), which further advanced interests in the utilization of true metallic gallium for 2D material applications.^[^ ^10^ ^]^ The structure of gallenene is stable down to the thickness of a single atom,^[^ ^15^, ^16^ ^]^ similar to graphene. In addition, single layer gallene has been fabricated through the interaction of liquid gallium between epitaxial graphene and SiC.^[^ ^17^, ^18^ ^]^ The existence of a stripe domain structure was demonstrated in gallium thin films grown on Si(111) via molecular beam epitaxy.^[^ ^19^, ^20^ ^]^ Furthermore, epitaxial bilayer gallenene on GaN (0001) can be produced using metal organic chemical vapour deposition.^[^ ^21^ ^]^ A theoretical study has demonstrated that Ga can form stable monatomic linear and zigzag chain structures that are metallic.^[^ ^22^ ^]^

The concept of free‐standing layer‐stacked 2D gallium crystals has been put forth, wherein self‐assembled layer structures are capable of exhibiting positional and orientational order with typical electro‐optical switching characteristics analogous to those observed in liquid crystal displays.^[^ ^23^ ^]^ However, van der Waals stacking of non‐centrosymmetric single‐element polar gallenene crystals in a supercooled liquid gallium (SLG) environment with switchable ferroelectric polarization has not been realized. Partly, due to fundamental limits such as the opacity of the liquid environment, which presents significant challenges to researchers. To bridge the existing gap between the polarization, piezoelectric, and nonlinear optical switching of SLG and its alloys, techniques such as transmission electron microscopy (TEM) and second harmonic generation (SHG) are essential for probing the internal structure of these liquids with precision.

Liquid metals (LMs) employ the fluidic transport of liquids while simultaneously enabling exceptional thermal and electrical conductivity.^[^ ^24^ ^]^ Ga‐based LMs are used in a diverse range of applications spanning microelectronics to soft actuators^[^ ^25^, ^26^ ^]^ and conductive inks.^[^ ^27^, ^28^ ^]^ LMs can be formed because of the supercooling effect in the pure form of gallium when heated above its melting point (29.8 °C) or in an alloy composition. The typical Ga alloys are eutectic gallium‐indium (75% Ga and 25% In by weight, 15.7 °C melting point) and gallium‐indium‐tin (68.5% Ga, 21.5% In, and 10% Sn by weight, −19 °C melting point).^[^ ^29^ ^]^ Furthermore, the characteristics of Ga‐based LMs are not limited to those of conventional liquids, as they also display exceptional properties such as anomalous density,^[^ ^30^ ^]^ phase separation,^[^ ^31^ ^]^ structural order,^[^ ^32^ ^]^ and chemical stability.^[^ ^33^ ^]^

SHG describes the phenomenon whereby a sinusoidal light wave of fundamental wavelength 𝜆 is converted to a second harmonic wave with a wavelength of 𝜆/2 upon interacting with a nonlinear medium.^[^ ^34^ ^]^ SHG imaging produces contrast based on the phase‐matching conditions; if the phases are equal between the fundamental wave and the second harmonic wave, the SHG intensity increases; conversely, mismatched regions produce little to no SHG signal.^[^ ^35^ ^]^ Furthermore, SHG intensity is influenced by the excitation geometry, enabling the technique to be employed, for example, in the study of 3D collagen structures within tissue^[^ ^36^, ^37^ ^]^ and the analysis of nanostructured metal compositions.^[^ ^38^ ^]^ In the case of metals, the SHG intensity is at its greatest when the excitation occurs along the crystal axis.^[^ ^39^ ^]^ This relationship has enabled the use of SHG for the study of the diverse compositions of metals, including gallium selenide,^[^ ^40^ ^]^ gold nanoparticles,^[^ ^41^, ^42^ ^]^ transition‐metal dichalcogenides (TMDC),^[^ ^43^, ^44^, ^45^ ^]^ nematic liquid crystals,^[^ ^46^ ^]^ and cadmium selenide nanowires.^[^ ^47^ ^]^

In this report, we employ microstructural characterization techniques, including transmission electron microscopy and nuclear magnetic resonance spectroscopy, to elucidate the structure of polar 2D gallenene flakes in a confined LMs environment. We obtained the stabilized 2D gallenene crystal through confinement and thermal annealing process. The abundant liquid metal provides a continuous air‐sealed hermetic environment that protects the polar 2D crystals from degradation. The nanocrystal terminations under investigation are single‐indexed gallium crystals with orientation a100, which also have the potential to self‐assemble and result in the formation of filaments.^[^ ^48^, ^49^, ^50^ ^]^ Subsequently, the nonlinear properties of the nanostructures are investigated through the utilization of nonlinear optical techniques based on SHG signal, with the objective of characterizing the non‐centrosymmetric polar response of the 2D stacked gallenene nanocrystals during angular, thermal, and electrical perturbation. The nanocrystals are embedded in the confined SLG films, which are sandwiched between two indium‐tin‐oxide (ITO) conducting glass slides that are coated with a polyimide alignment layer. This polyimide layer is used in liquid crystals research to align liquid crystal mesogenes.^[^ ^51^, ^52^ ^]^ Moreover, two‐terminal measurements reveal reversible ferroelectric polarization switching in the confined SLG film. First‐principles (density functional theory) DFT calculations further confirm the structural stability of the multilayer planar gallenene and predict an intrinsic piezoelectric response based on the calculated piezoelectric tensor.

## 2 Results and Discussion

### 2.1 Atomic and Electronic Structure of SLG

**Figure 1:**
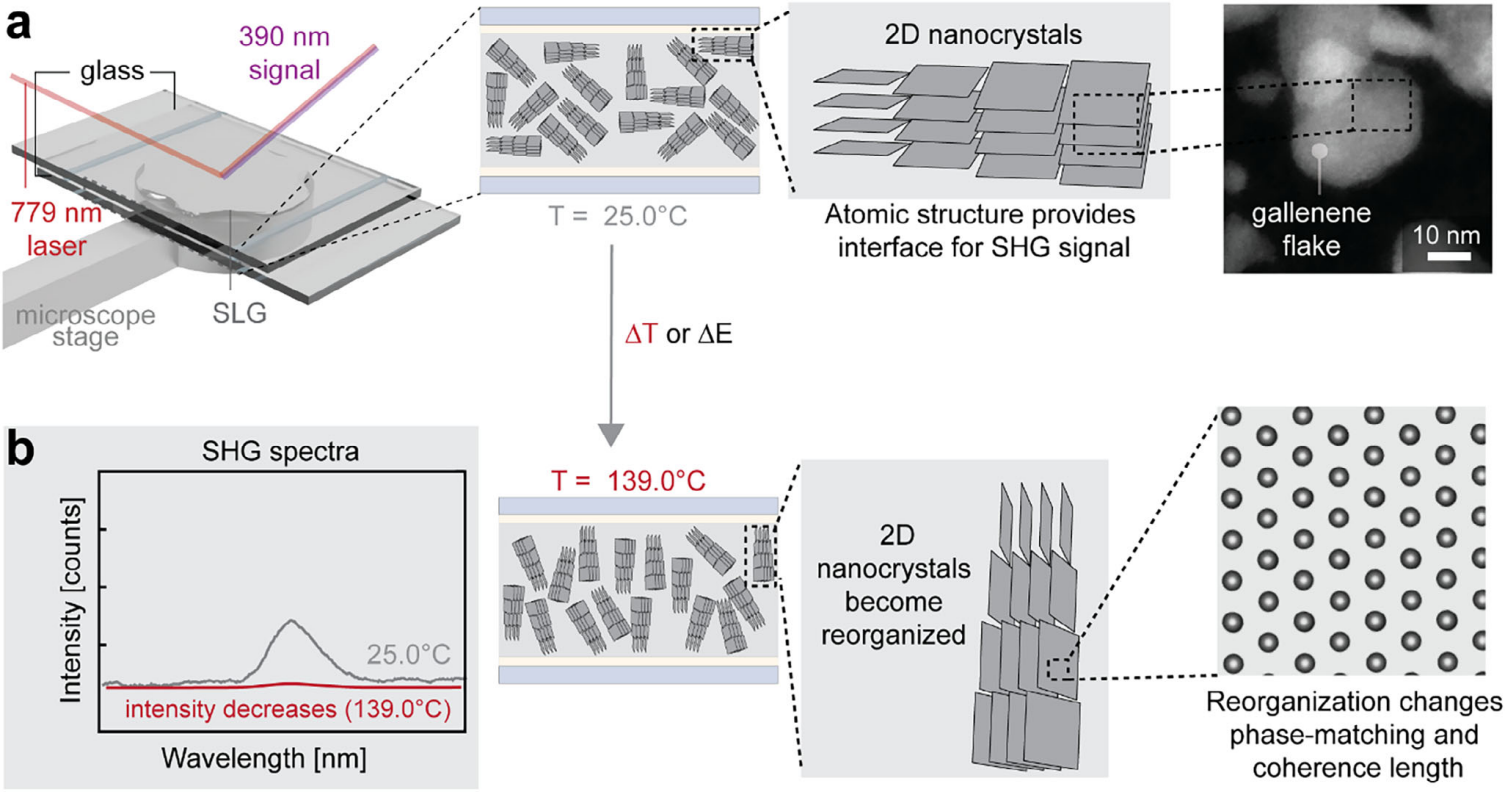
Structure of gallenene and complex anatomy of supercooled liquid gallium. Mechanism for electrical and thermal perturbation. a) Illustration of hypothesized interaction of SHG response with SLG in linearly polarized light showing that thermal perturbations could align the 2D nanocrystals, allowing for an increased SHG medium at either temperature or electrical fields. An example HAADF image of gallenene flake sandwiched between two graphene layers, as depicted in (a) (far right microscope image). b) Structural reorganization of gallenene nanocrystals in the SLG leading to an intensity change in SHG signal as a result of thermal or electrical perturbation.

We begin the study of polar nanocrystals in SLG by employing the SHG technique, which requires a non‐centrosymmetric medium for generating the frequency doubled optical signal (**Figure** **1**a). To understand the compatibility of SLG with the SHG imaging technique, we investigated the microstructure of SLG through linearly cross‐polarized light microscopy (PLM) in reflection (Figure S1, Supporting Information). Our analysis shows that the observed nanocrystals and fibres appeared to be twisted like a twist grain boundary.^[^ ^53^ ^]^ Through further characterization and using atomic force microscopy, we verified the spontaneous self‐organization of the nanocrystals. We visualized the broken helical filament structures with layers parallel to the substrates from the thin layer of SLG at room temperature in between polydimethylsiloxane (PDMS) substrate and an acrylic‐coated glass (Figure S2, Supporting Information). To obtain more information on the microscopic spatial arrangement of the fibre structure, we conducted scanning electron microscopy (SEM) imaging of a slowly cooled SLG droplet between a cleaned glass and PDMS surface. Our observation revealed highly oriented superstructures of helical filaments geometry (Figure S3a, Supporting Information). These microstructure images indicate that the structure of SLG has twisted filaments, providing the characteristic twist feature of SLG (Figure 1a). In the confinement configuration in Figure 1b, the unordered filaments are organized to alter the signal intensity during measurement with different perturbations, such as electrical or thermal changes.

To study the nanocrystal behavior at the atomic level, we encapsulate SLG by sandwiching it between two monolayer graphene sheets on a perforated SiN_x_ membrane (Figure 1a). The scanning transmission electron microscopy (STEM) images of the graphene‐encapsulated gallenene provide evidence of multilayer gallenene flakes with a 100 orientation. For further verification, high‐angle annular dark‐field (HAADF) images of gallenene between the two graphene layers are shown in Figure 1a. The Ga‐L edge detected by electron energy loss spectroscopy (EELS) confirms the presence of gallium and its metallic state (Figure S3b,c, Supporting Information). The EELS spectra were obtained from the marked area in Figure 1a.

**Figure 2:**
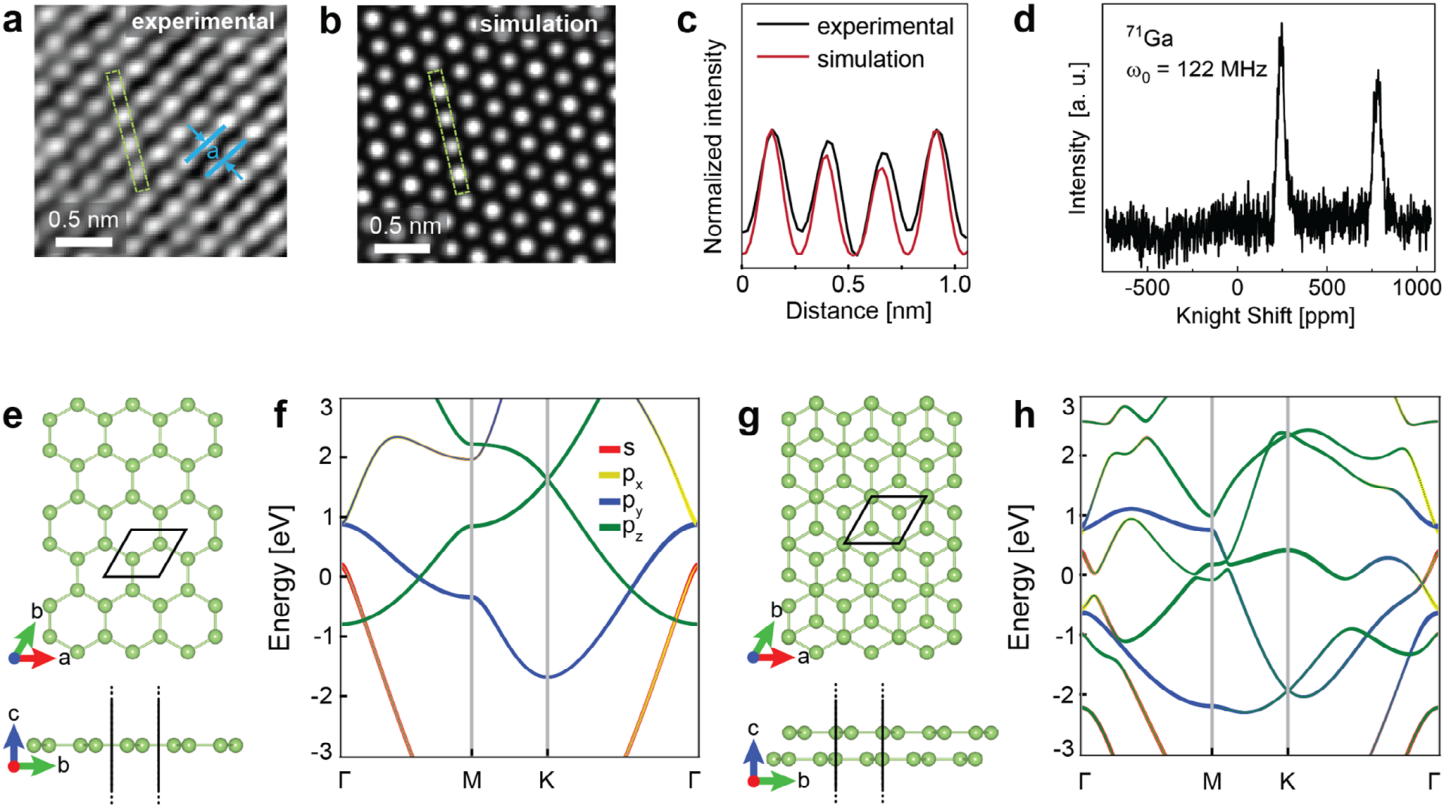
Assessing the atomic structure of SLG. a, b) Atomic resolution HAADF image of multilayer gallenene and the corresponding simulated HAADF images of 12 layers, respectively. The lattice spacing is denoted as *a*. c) Intensity profile recorded on the experimental and simulated HAADF images. The intensity profiles are recorded along the dashed green rectangle in (a and b). d) ^71^Ga NMR signal of SLG at room temperature. The peak splitting of the ^71^Ga isotope is obtained in a micro‐capillary filled with SLG. The resonance frequency ω_*o*_ of ^71^Ga is 122 MHz. e,f) Top and side views of the optimized crystal structures of monolayer and bilayer a100, left, respectively. The orbital projected band structure of monolayer a100, highlighting the Dirac cone at the K point above the Fermi level, originating from the p_z_ orbitals. The Dirac cones along the Γ‐M or Γ‐K directions, near the Fermi level, are formed by the p_y_ band situated above the p_z_ band in (e). The orbital projected band structure of bilayer a100, where the contribution near the Fermi level predominantly arises from p_z_ orbitals in (f).

To determine the atomic structure of the layered structures, we compared the atomic resolution HAADF image of gallenene (**Figure** **2**a) and the corresponding simulated HAADF image of multilayer gallenene (Figure 2b). In HAADF imaging, the intensity scales approximately as Z^1.6^ for isolated atoms and exhibits an approximately linear dependence on specimen thickness for a constant atomic number (Z).^[^ ^54^, ^55^ ^]^ In Figure 1a, the bright regions correspond to the gallenene flake, owing to its higher atomic number and greater thickness, while the darker regions represent areas of bare graphene, where no flake is present.

From the high‐resolution experimental results obtained, the a100 orientation of multilayer parallel stacked gallenene appears to be the stable termination on graphene. The intensity profiles recorded along the dashed green rectangle in Figure 2a,b show that the experimental and simulated HAADF images are in good agreement (Figure 2c). The experimental data combined with simulations further confirms that the Ga structures are single‐indexed with ≈12 layer stacks of 2D gallenene, similar to previously reported studies for atomically flat terraces^[^ ^56^ ^]^ or exfoliated monolayers.^[^ ^14^ ^]^ Moreover, the Fourier transform of the Gaussian filtered HAADF image clearly shows the hexagonal ring structure of the gallenene a100 structure (Figure S4, Supporting Information). The lattice spacing of the AB stacked gallenene a100 is measured to be 0.22 ± 0.01 nm based on the average of multiple measurements taken directly from real‐space, atomic‐resolution HAADF‐STEM images. This measured lattice parameter agrees with the reported literature values of the bilayer gallenene structure.^[^ ^10^, ^14^ ^]^ Since Ga exhibits strong polymorphism with different crystal modifications, we investigated the possible dipole/quadrupole coupling of gallenene structures in SLG using nuclear magnetic resonance (NMR) spectroscopy.^[^ ^57^ ^]^ We observed a peak splitting at room temperature in capillary confinement (Figure 2d). The ^71^Ga resonance frequency in the SLG is 122 MHz.^[^ ^58^ ^]^ The observed peak splitting is consistent with previous studies of liquid Ga in a confined porous matrix.^[^ ^57^, ^59^, ^60^ ^]^ In another view, the known polymorphism of Ga leads to different crystal modifications, where the initial condition of the liquid state plays a crucial role. The different precursors of the liquid state could contain quasi‐clusters leading to the different components of the NMR signals.^[^ ^59^, ^60^ ^]^

Monolayer and bilayer‐stacked gallenene structures were investigated by DFT calculations (Figure S5, Supporting Information). We obtained a relaxed atomistic model from monolayer to bilayer AB stacking. The bulk α‐Ga structure crystallizes in the Cmca space group, with lattice constants from fully optimized structures determined as a  = 0.459 nm, b  =  0.776 nm, and c  =  0.459 nm, which is in good agreement with experimental data.^[^ ^14^ ^]^ DFT analysis reveals that the a100‐oriented gallenene monolayer has a planar hexagonal crystal structure with a Ga–Ga distance of 0.262 nm, consistent with previous reports.^[^ ^10^, ^14^, ^61^ ^]^ It displays metallic electronic characteristics (Figure 2e,f). We also modelled a freestanding bilayer gallenene structure, which consists of AB‐stacked planar a100 monolayers with bulk lattice parameters. Based on our experimental results, we considered AB‐type stacking, with the interlayer distance set to the experimentally measured lattice spacing of 0.22 nm. The DFT calculated bilayer structure agrees with the experimental results. The electronic band structure of the bilayer a100 remains metallic (Figure 2g,h). Additionally, the trilayer a100 structure was analysed with both ABA and ABC type stacking configurations, and the ab initio calculations indicate that ABA‐type stacking is energetically more favourable (Figure S6a–d, Supporting Information). Moreover, the 12‐layer structure was also modelled, considering both ABA…B and ABCA…C stacking configurations. The results indicate that the ABA…B stacking is energetically the most favourable (Figure S6e–h, Supporting Information). We obtained planar structures in all the relaxed atomistic models set to the experimental lattice spacing of 0.22 nm. We further investigated symmetry breaking of the gallenene structure through first‐principles calculations. Specifically, we calculated the piezoelectric tensor (*e_ij_*) for AB‐stacked multilayer Ga(100) using density functional perturbation theory (DFPT). For 2D materials, the relaxed‐ion piezoelectric tensor is expressed as:

(1) $$e_{ij}=e_{ij}^{el}+e_{ij}^{ion}$$

where $e_{ij}^{el}$ and $e_{ij}^{ion}$ denote the electronic and ionic contributions, respectively. The raw piezoelectric data are provided in the Supporting Information section. Our DFT results are consistent with the experimental observations. All multilayer Ga(100) structures exhibit non‐zero in‐plane polarization; with increasing layer number, an out‐of‐plane component emerges and becomes pronounced in the 12‐layer system. This piezoelectric response originates from the lack of inversion symmetry in the non‐centrosymmetric AB stacking of Ga(100) layers.^[^ ^62^, ^63^ ^]^ These findings suggest that Ga(100) multilayers are promising candidates for piezoelectric applications. The multilayer Ga(100) exhibits P1 symmetry corresponding to the C1 space group. We obtained piezoelectric tensor coefficients on the order of 10^−10^ *C*/*m*, consistent with the spontaneous polarization measured in 2D single‐element bismuth monolayers (*Ps*  =  0.41  ×  10^−10^ *C*/*m*).^[^ ^3^ ^]^ It should be noted that a perfectly hexagonal, AB‐stacked Ga(100) bilayer belongs to the centrosymmetric space group P‐3m1. Due to the presence of inversion symmetry in this space group, piezoelectricity is not supported by symmetry.^[^ ^62^, ^63^ ^]^ Consequently, this symmetric configuration was excluded from our analysis.

The broken polar symmetry of the gallenene structure allows the determination of the structural order and polarity of the SLG fibres through SHG. In polar metals, second harmonic generation has been shown to be a promising imaging technique with the highest possible susceptibilities in metals approaching 10 nm V^−1^.^[^ ^11^ ^]^ Therefore, we proceed with the investigation of gallenene flakes using second harmonic generation imaging to determine how fibre orientation is affected with respect to various perturbations.

### 2.2 Nonlinear Response of SLG

SHG describes the phenomenon where two photons of the frequency ω, upon interacting with a nonlinear medium, are converted to a single photon with a doubled frequency 2ω.^[^ ^64^ ^]^ This interaction of light with a medium results in a polarization defined by the equation: $P=\varepsilon_{0}\cdot \left[\chi^{\left(1\right)}\cdot E+\chi^{\left(2\right)}\cdot E\cdot E+\chi^{\left(3\right)}\cdot E\cdot E\cdot E+\cdots \right]$. Where ɛ_0_ is the vacuum permittivity, and E is the incident electric field. When the vacuum permittivity is distributed, we are left with the summation of several terms. The term ɛ_0_ · χ^(2)^ · *E* · *E* gives rise to second harmonic generation, or SHG, including the second‐order polarizability tensor χ^(2)^, which is only non‐zero for non‐centrosymmetric materials. This non‐centrosymmetric structure can be given at the interface of two different materials, leading to surface SHG. In our measurements, however, we can diminish and even eliminate the SHG signal through electrical perturbation, which will be discussed in detail later. This leads to the conclusion that the main SHG in SLG is not due to a surface effect, as a change of the response in surface SHG in such a way would mean a removal of the gallium‐glass or gallium‐crystal interface for our measurement. The behavior of the signal for varying excitation angles can be investigated, as the intensity of the SHG signal depends on the relative position between the excitation electric field and the crystal axis. Individual fibres with a statistical distribution should show different SHG responses due to the traversed distance of the incident light through the crystal. This is due to the higher amount of constructive interference when the phase matching condition allows SHG.^[^ ^65^, ^66^ ^]^ In other words, when the excitation electric field matches the individual orientation of the fibres, SHG maxima will be observed, and SHG minima for electric field cross‐oriented to the fibres.

### 2.3 Periodic SHG Signals

**Figure 3:**
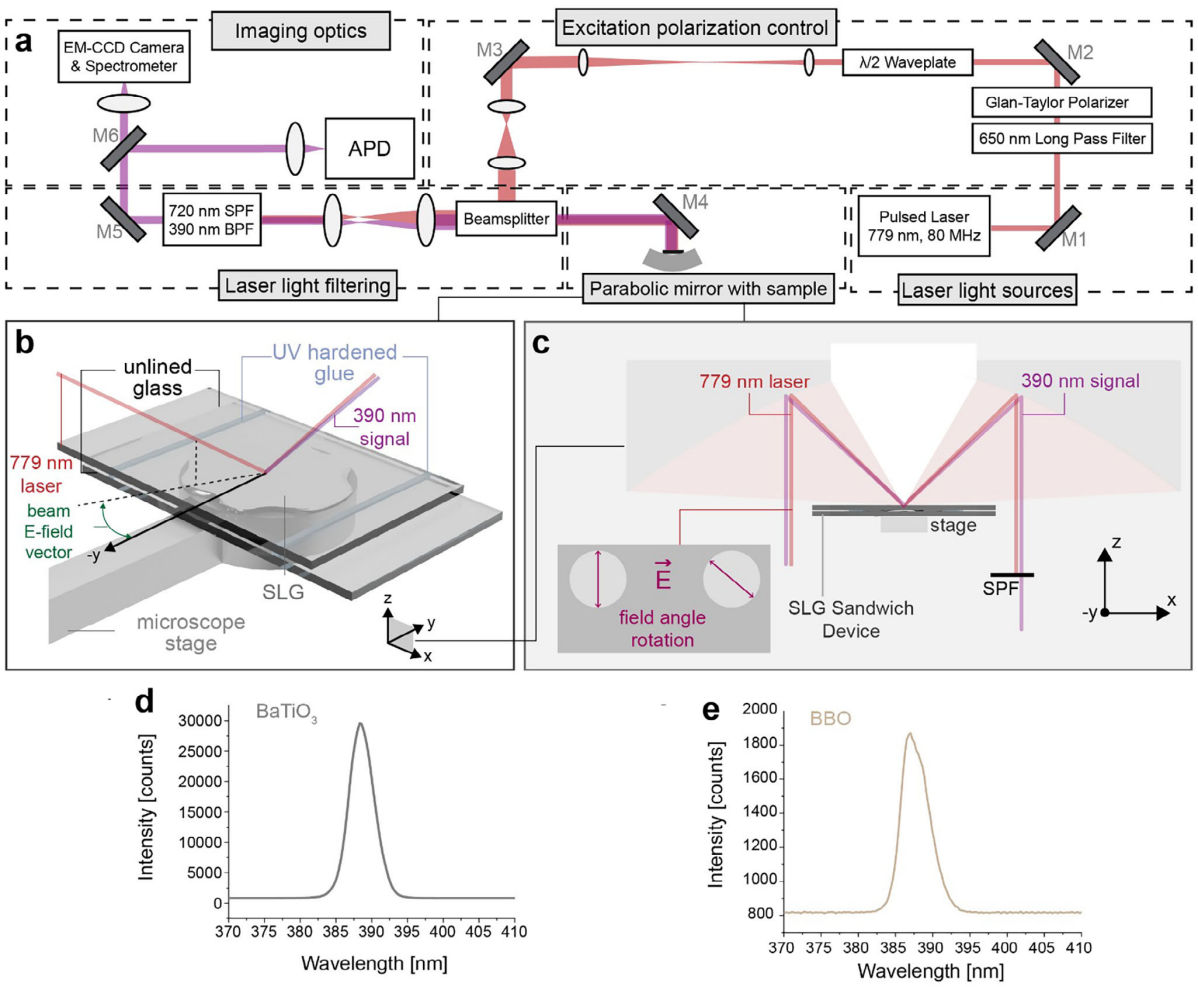
Custom‐built SHG microscope and SLG sandwich device setup. a) Custom‐built confocal microscopy SHG setup with a static sample stage and a linearly polarized pulsed laser (λ = 779 nm, repetition rate = 80 MHz). It has five different sections, starting with laser light sources and ending with optics for imaging. b) Schematic of the SLG in a sandwich device placed in the focus of the parabolic mirror from (a). c) The configuration of the sandwich device showing the field angle rotation. The angle of the incident electric field polarization is rotated via a 𝜆/2 waveplate from (a). The SHG signal is separated from the fundamental laser light by a short‐pass filter (SPF). d,e) SHG spectra for BaTiO_3_, and β‐BaB_2_O_4_, respectively, as a reference for the functionality of the self‐built SHG microscope. The laser power was reduced from 8 mW (SLG) to 5 mW (β‐BaB_2_O_4_), or 1 mW (BaTiO_3_), and the acquisition time was reduced from 30 s (SLG) to 0.1 s (β‐BaB_2_O_4_, BaTiO_3_) due to the high SHG intensity of these materials.

Using a custom‐built parabolic mirror confocal microscope in **Figure** **3**a,^[^ ^67^ ^]^ we analysed the SLG to observe the SHG through the gallium as a non‐linear medium (Figure S7a, Supporting Information), which allowed us to identify the amount of SHG response (Figure S7b, Supporting Information).

**Figure 4:**
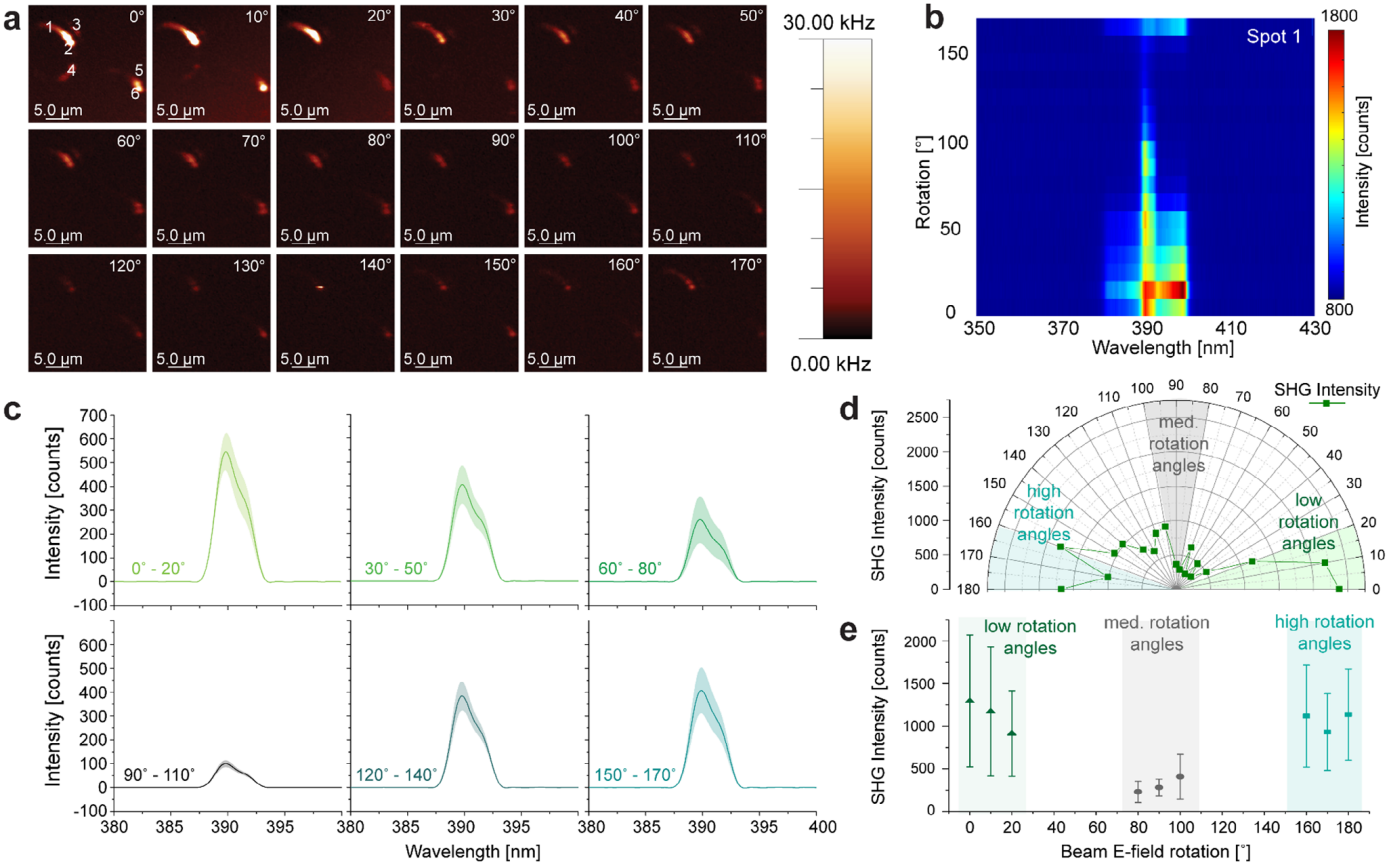
Field angle rotation SHG measurements of SLG. a) Intensity images of the SLG at 18 different angles show the effect of various rotations on the SHG signal intensity. b) Pseudocolor plot of point 1 shown (a) across both wavelength and rotation. c) Average over three points of the SHG of the SHG integral for different field angle rotations from 0 to 180 degrees. d) Representative polar histogram of Spot 1 in Figure S8d showing the change in SHG intensity and the 180‐degree periodicity of the maximum intensity of the SHG signal in the SLG samples. e) SHG intensities averaged over five spots that are excited with electric fields of low, medium, and high rotation angles. The colour codes for the lightly shaded bars are correlated with those in (d). To achieve averaging of multiple spots with individual preferred orientations, the maximum SHG signal was set to 0°.

We accomplished geometrical orientation changes of the supercooled liquid gallium sandwich‐devices (SLG‐SD) through imaging a static sample (Figure 3b,c) with an angularly rotating beam electric field vector (**Figure** **4**a–c). In contrast to the thermal and electrical perturbations to be discussed later, the SLG‐SD remains stationary and physically unchanged here. To demonstrate the custom‐built SHG microscope works for conventional nonlinear samples we measured two common and previously published materials, including, β‐BaB_2_O_4_, and BaTiO_3_, and demonstrate they both exhibit a high SHG response (Figure 3d,e) and showcase petals from angular responses (Figure S8a, b), which are consistent with previously published data on β‐BaB_2_O_4_,^[^ ^68^, ^69^ ^]^ and BaTiO_3_.^[^ ^70^, ^71^ ^]^ In scans of the single crystal, a strong response to this excitation direction can be seen (Figure 4a). We observed a significant change in SHG intensity about the vector rotation angle, θ (Figure 4b,d), indicating a favourable excitation geometry is present in the SLG (Figure 4b,d). For individual spots, we observed a significant shift between aligned and perpendicular vector rotation, indicating a preferred orientation due to a distinct geometry at a single spot (Figure 4e; Figure S9a, b, Supporting Information). Long traverse distances through the gallium crystals show comparatively high SHG response, with an SHG minimum expected at 90° rotation offset from the maximum due to the lower traverse distance. This resulted in an up to 10.4 fold larger SHG signal for the aligned beam. This is further influenced by the phase matching condition for SHG. This behavior indicates that the SHG dipole moment of the fibres is parallel to the incident field.^[^ ^45^ ^]^

SHG intensity scans of the SLG‐SD revealed that multiple fibres with different orientations were present in the same region of interest (Figure 4a). Their SHG intensity is influenced in a similar way for all of them. When the beam electric field is rotated over the SLG‐SD, the SHG response changes, as it depends on the orientation of the electric field to the crystal axis. Our experiments demonstrate this periodic behavior when the electric field is rotated stepwise from 0° to 360° (Figure S9a–d, Supporting Information) with a linearly polarized laser (Figure S20a, Supporting Information). As can be seen in Figure S9d, the SHG response pattern repeats similarly after a 180° rotation with a point mirror symmetry about the centre of the polar plot. This is due to the focal field of the linearly polarized laser rotating about the optical axis when the beam electric field is rotated by the λ/2 waveplate, which leads to a 180° periodicity for the excitation electric field direction (Figure S20a, Supporting Information) matching fibre orientation.

This trend is consistent with other transversely isotropic structures, including TMDCs^[^ ^44^, ^45^ ^]^ and ferroelectrics,^[^ ^72^ ^]^ highlighting the preferred orientations of these liquid gallium microstructures. This highlights the ability of SHG measurements to not only show the structure of these SLG crystals, but also their orientation in the liquid gallium medium. In previous LM studies, a channelled glass plate has allowed the alignment of the LM crystals.^[^ ^23^ ^]^ If similarly possible in the liquid gallium, it would provide a preferred polar orientation to allow a uniform excitation geometry of the SLG, enabling the possibility of reliable switching between minimum and maximum SHG optical response using the electric field vector of the beam. The SHG tunability of SLG could potentially be employed in metamaterials applications.

### 2.4 Thermal Perturbations Indicating Phase‐Change

**Figure 5:**
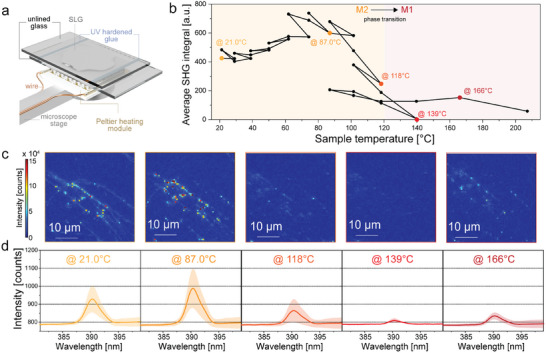
Thermal perturbation of SLG. a) SHG intensity measurement setup of an SLG sample on a Peltier hot plate. b) Average SHG area over different sample temperatures with insets of points corresponding to the specific scan areas shown in (c) and spectra shown in (d). The transition between phase M2 and M1 at 120 °C is taken from previous work by Yunusa et al.^[^ ^23^ ^]^ c) Scan images of the SHG response starting from 21 °C up to 207 °C at five points highlighted in colour in (b). d) The average SHG spectral intensity at five highlighted points shown in (b), with the intensity decreasing to nearly zero with low deviation at 139 °C.

We used the same custom‐built confocal optical setup in Figure 3a to image the SLG‐SD attached to a thermocouple device (**Figure** **5**a). This thermal perturbation follows a different mechanism than the previously described angular electric field vector perturbation. The temperature increase is expected to cause physical changes in the sample structure due to thermal expansion and phase transition, while keeping the sample orientation and beam rotation fixed. This phase transition should be visible in a SHG response change, as the crystal‐lattice and orientation will be changed, and therefore, the phase matching is changed. We found a clear dependency of the SHG intensity on the temperature of the SLG sample (Figure 5b). In a lower temperature range (23–80 °C), the SHG intensity increased steadily with a maximum found ≈80 °C (Figure 5b,d). The increased SHG response may be attributed to thermal expansion and modification of the SLG refractive index due to heating, which can cause a changed interference result for SHG following the phase matching condition.^[^ ^73^, ^74^ ^]^ This assay is robust to cyclic temperature stress, and in this low temperature range, the physical structure of the sample appears to be unaffected, as the scanned images show no significant changes. In this range, repetitive temperature increases and decreases show a reproducible SHG response. When the SLG reached the median range of temperatures (80–120 °C), we saw a steep drop in the SHG intensity, which decreased to 50% of the spectra recorded at room temperature (Figure 5b,d). Finally, at 140 °C, the SHG signal disappears completely for one cycle, which is accompanied by a clear visual change to a partial liquid state (see Video S1, Supporting Information) and a drastic change in the scanned image of the sample (Figure 5b–d). The formation of bubbly domains was reported in previous studies on similar devices.^[^ ^23^ ^]^ In addition, the viscosity of SLG decreases as the temperature increases from room temperature to the phase transition temperature.^[^ ^75^ ^]^ Newly found spots in the scanned image emitted mostly luminescence, and the SHG signals obtained were very low or zero (Figure 5b,d; Figure S10, Supporting Information). This behavior indicates a phase change in the SLG because the local structure must be altered, which has been previously described as a shift from the MP to the M2 and M1 phases in 1,3‐dioxane (DIO) and SLG.^[^ ^23^, ^46^ ^]^ We then cyclically decreased the temperature, resulting in the reappearance of the previously found scanned image and SHG signal. Although the SHG signal intensity is much weaker than for the same temperatures measured previously, it increases steadily until ≈80 °C is reached. This indicates that during the phase transition back to the M2 phase, the lattice and structural arrangement are not reconstructed exactly to the previous state. This is true for the entire temperature range (up to 200 °C) that is available for the heating device. The temperature increase before the phase change affects the SHG signal, with the SHG signal disappearing after the phase change, similar to the behavior common to ferroelectric liquid crystals.^[^ ^46^, ^76^ ^]^ Increasing the temperature further beyond this phase transition temperature leads to a weak reappearance of the SHG signal, indicating that the second phase, which has appeared due to structural rearrangement in the SLG at higher temperature, also exhibits weak nonlinear optical properties. Similarly, when observed under linear cross‐polarized optical microscopy, the intensity of the SLG texture reappeared above 120 °C during heating due to thermal agitation and structural rearrangement.^[^ ^23^ ^]^ This indicates that the nanocrystals in SLG maintain their structural order even at high temperature, which is supported by the scanned image at 166 °C.

### 2.5 Polarity Switching in Electrical Perturbations

**Figure 6:**
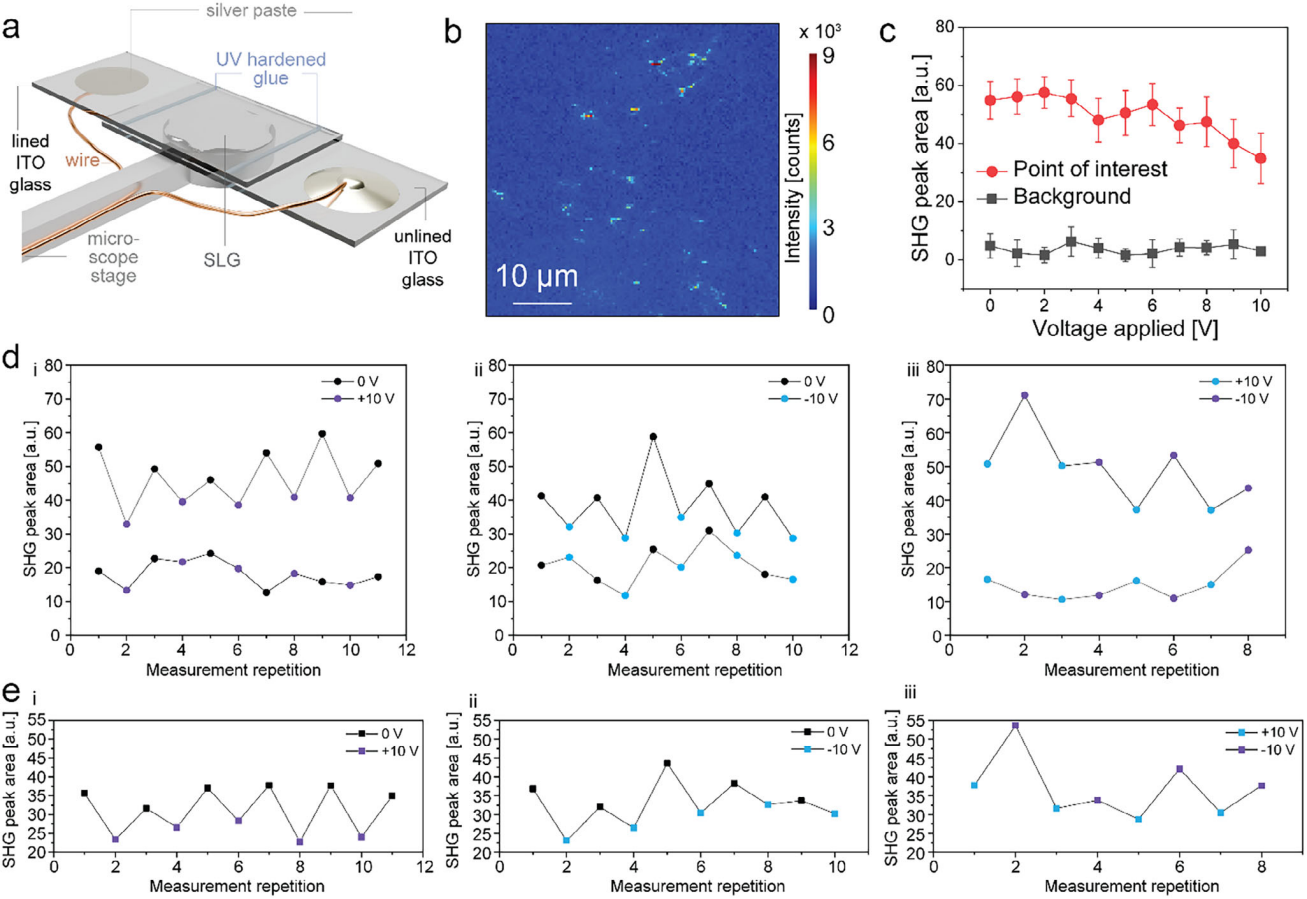
Electrical perturbation of SLG. a) Schematic showing a new SLG sandwich device with wires for electrical charging. b) Scan image of the SLG during electrical perturbation at +10 V. The applied voltage results in a visible deviation from the initial filament structure. c) SHG intensity difference between the background and the averaged points of interest during electrical perturbation. d) SHG intensity switching for exemplary highly responsive spots (upper graph) and statistically responsive spots (lower graph) for (i) 0 V and +10 V, (ii) 0 V and ‐10 V, and (iii) +10 V and ‐10 V. e) Average SHG response of highly response spots (*n* = 6 (i), 4 (ii), 5 (iii)) for (i) 0 V and +10 V, (ii) 0 V and ‐10 V, and (iii) +10 V and ‐10 V.

Finally, we imaged the SLG‐SD with electrical bias (**Figure** **6**a) to test electrical perturbations using the same custom confocal SHG setup (Figure 3a). Prior to electrical perturbations, the prepared SLG samples were pre‐aligned by applying a square waveform 16 V_pp_ (peak‐to‐peak) or DC field. The pre‐alignment effect induced reorientation of polar order in gallenene nanocrystals. Note that the pre‐alignment field strength depends on the sample thickness, and the occurrence of different polarities due to fibre orientation is possible. Our observation indicates a non‐zero SHG signal for the pre‐aligned condition. We found that the susceptibility to electrical perturbation differs depending on the sample orientation studied. In a few experiments, the physical structure of the sample changes significantly in orientation, which can be observed visually by the SHG scan of the stationary sample as the applied voltage is adjusted (Figure S11, Supporting Information). The structural reorientation of the sample is the result of the different polarity of the alignment. Although these significant changes were observed with certainty, they can be restored to the previous state when the electrical perturbation is applied in the opposite bias, but in most cases not to the exact correlation of the electrical perturbation. The response of the SHG is directly related to the orientation of the average long axes of the structures, which is defined as a director (**n**). This indicates that electrical perturbation can lead to significant structural reorientation in the SLG to either align or misalign the axes of the structures with the incoming fundamental wave.^[^ ^77^ ^]^ Such rearrangement of molecules or fibres is common in liquid crystals due to the field application.^[^ ^78^ ^]^ Permanent damage to the SLG‐SD is observed when voltages of more than +10 V or −10 V are applied, which is indicated by an irreversible disappearance of the SHG response (Figure S11, Supporting Information) and might be attributed to hydrodynamic drift due to Joule heating in the SLG. Within a reasonable range, however, the observed changes in SHG are much smaller than those of thermal or geometrical changes in the structure, similar to that of DIO.^[^ ^46^ ^]^ This smaller effect on the electrical intensity could be due to the slow response of gallenene nanocrystals to realign during electrical perturbation, as the polarity of the field plays a role in the orientation. Therefore, after an electric field is applied, they all experience an electric field force, but only some physically shift, as the electrical stimulation of the sample could be more localized compared to the thermal signal.^[^ ^79^ ^]^ Experimentally, the crystals therefore rotate into or out of the focal plane, leading to a change in SHG response, which we compare between multiple forced orientations, i.e., different biases applied. The rotation of the crystals either leads to parallel or perpendicular alignment to the incoming light. We note that the strongest SHG response here is biased to the 0 V case with no perturbation, as the regions of interest on which the experiments were conducted were searched with no electrical bias applied. This indicates that the incoming polarization is parallel to the long axis of the crystal in focus. Thus, the measured area is biased to show a strong SHG signal in the 0 V case, and electrical perturbations are expected to decrease the SHG response. Compared to the background, where the weakest signals are observed in the scanned image, spots of higher intensity are more sensitive to electrical perturbation (Figure 6b,c),^[^ ^80^ ^]^ indicating that orientation changes are likely to occur depending on the initial **n** direction and polarity.

We investigated SHG switching during electrical perturbations, which leads to optical switching of the SHG intensity, as shown in Figure 6d,e (Figure S11b, Supporting Information) for multiple regions of interest. When the applied voltage was switched between 0 and −10 V in multiple cycles, the SHG signal, on average, follows the trend of two presumed orientations switching repeatedly (Figure S11b, (i) Supporting Information). The characteristics of this differ between spots, as some spots exhibit SHG intensity switching over the full range, while some spots only partially follow the trend (Figure 6d (i)). Similar results are reproduced when the applied voltage is switched between 0 V and +10 V (Figure 6d,e (ii); Figure S11b (ii), Supporting Information) and between −10 V and +10 V (Figure 6d,e (iii); Figure S11b (iii), Supporting Information). We can differentiate between spots that clearly follow the switching behavior and spots that have a statistical SHG response (Figure 6d). As this is not an ensemble measurement, it is expected that individual spots d not show perfectly identical behavior due to their initial orientation parallel or perpendicular to the incoming polarization^[^ ^77^ ^]^ and their individual local chemical environment. The ability to consistently manipulate the response with polarity shifts allows for rapid electrical switching, which is a common use of liquid metals in microelectronics.^[^ ^81^ ^]^

Although all spots show individual behavior, the reliable switching trend of the average SHG intensity across all points of interest persists in all experimental cases (Figure S11b, Supporting Information). We found that the SHG switching behavior of active spots (Figure 6e) is preserved even when averaged with the non‐switching spots (Figure S11b Supporting Information), which indicates that parts of the sample follow the switching behavior consistently, while others behave statistically. Those that show switching behavior express SHG changes in the same way, i.e., the same oriented state shows higher SHG intensity for all that show switching behavior. The polarity of the field dictates the individual behavior for single spots due to variations in organization, structure, and order. In other words, we can control the magnitude of the intensity by perturbing the structural properties with positive and negative voltages. To support the non‐centrosymmetric polar breaking effect in gallenene nanoflakes, we measure the spontaneous polarization in the layered 2D crystals using the polarization current reversal method commonly used to measure spontaneous polarization of ferroelectric liquid crystals.^[^ ^82^ ^]^ Due to the liquid crystal behavior of SLG and fluidity, the current reversal technique is suitable for the spontaneous polarization experiment. This approach complements the SHG observation in SLG and agrees with the literature studies on SHG signal detection in liquid crystal phases.^[^ ^46^, ^76^, ^83^ ^]^

### 2.6 Ferroelectric Response with PFM

**Figure 7:**
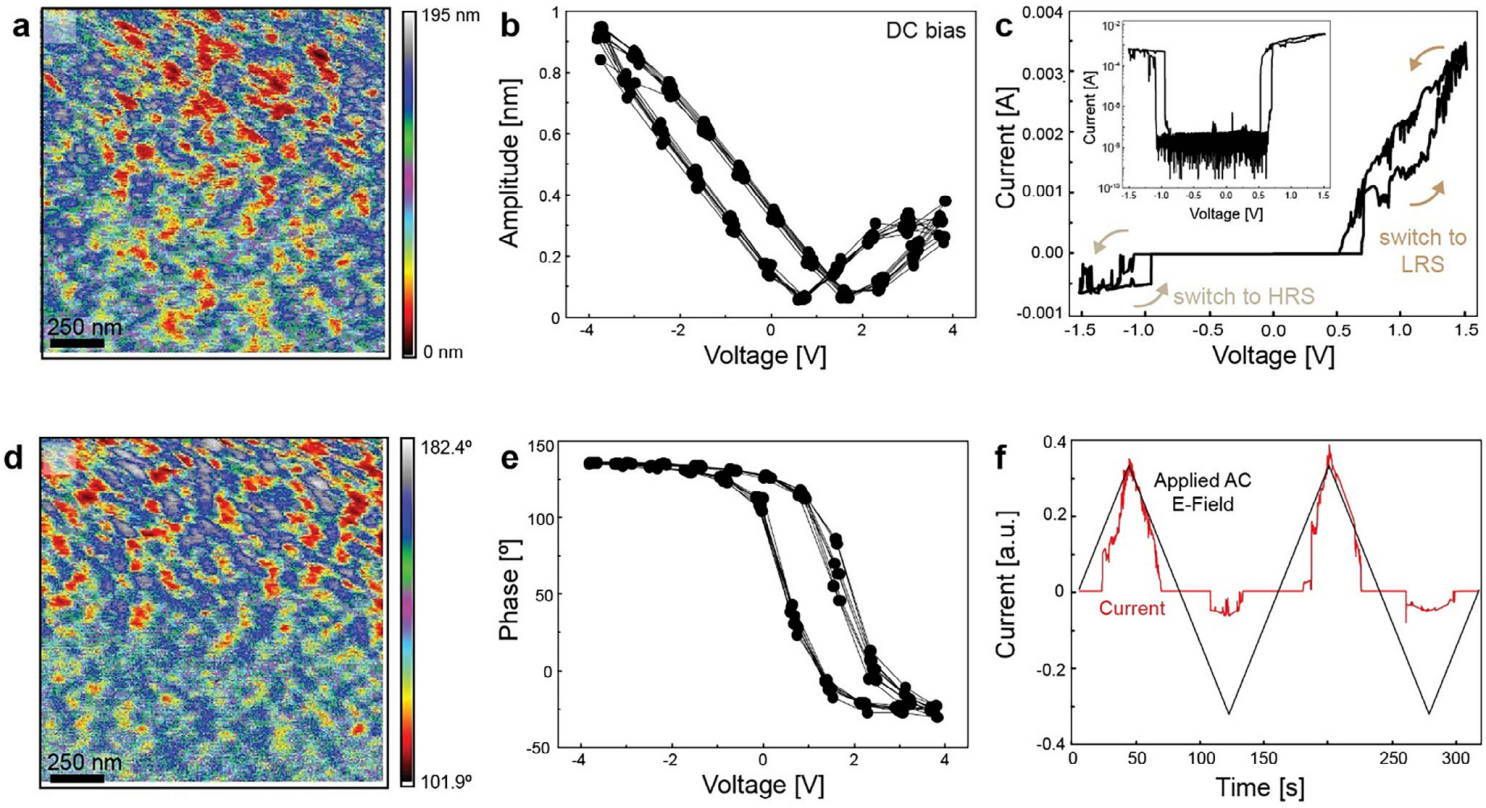
Piezoresponse force microscopy and device switching. a,d) ON‐field amplitude and phase images of ferroelectric SLG film. b,e) Typical ferroelectric butterfly shape and PFM phase measurement at room temperature exhibiting clear hysteresis effect by applying DC bias of ±4 V. c,f) Typical *I–V* characteristics of an ITO/SLG/ITO device showing bipolar switching effect behavior. The arrows in (c) represent the switching from HRS to LRS during the sweep. Switching current and voltage superimposed with time for 2 sweeping cycles (f).

To investigate the ferroelectric response in SLG, we obtained a hysteresis loop in ferroelectric SLG nanocrystals. We measured voltage‐dependent amplitude and phase using piezoresponse force microscopy (PFM) on SLG samples prepared by drop‐casting a suspension of particles (Figure S12, Supporting Information). The samples used for the measurement are a dense 50 µm‐thick layer of SLG on ITO glass with a defined geometry of 1 cm diameter. **Figure** **7**a,d displays the corresponding ON‐field amplitude and phase images. The typical ferroelectric butterfly shape and clear hysteresis are shown in Figure 7b,e. The corresponding well‐defined butterfly loops of the PFM amplitude signal and the phase were recorded using resonance‐enhanced PFM mode by applying an AC electric field superimposed on a DC triangle saw‐tooth waveform at a resonance frequency of 305 kHz (see Method for details and Figure S13, Supporting Information). The indication of the typical butterfly loop of amplitude signal and the 180° switching of phase signal confirm the ferroelectric polarization switching in SLG. The obtained PFM amplitude and phase results are consistent with the established ferroelectric materials in the literature, including 2D ferroelectric materials.^[^ ^84^, ^85^, ^86^, ^87^, ^88^, ^89^ ^]^

The butterfly loop shifts slightly toward positive voltage at zero applied voltage due to differences in Schottky barrier height between the PFM tip, SLG, and ITO substrate.^[^ ^90^ ^]^ In addition, the asymmetric appearance of the butterfly loop and hysteresis toward the positive voltage direction is the result of pre‐poling at +3 V.^[^ ^91^ ^]^ Furthermore, the ferroelectric switching effect was further verified using a 3‐electrode configuration in an electrolyte. The SLG films covering a 1 cm diameter active area were directly exposed to electrolyte while the rest of the ITO surface was isolated by Kapton tape (Figure S12, Supporting Information). The electrolyte consists of phosphate buffered saline (PBS). Cyclic voltammetry (CV) revealed distinct ferroelectric switching peaks in the bipolar current‐voltage loop, resembling those found in BaTiO_3_^[^ ^92^ ^]^ and ferroelectric liquid crystals^[^ ^83^, ^93^ ^]^ as compared in Figures S14 and S15 (Supporting Information),^[^ ^94^ ^]^ unlike the un‐poled sample^[^ ^95^, ^96^ ^]^ (see Figure S16, Supporting Information).

The pre‐poling was achieved at room temperature by applying −3 V versus Ag/AgCl, which aligns the domains within the ferroelectric SLG, creating a net polarization in the direction of the applied field. The combined PFM hysteresis loop and the bipolar current‐voltage loop corroborate the ferroelectric polarization in SLG that aligns with the typical ferroelectric profile of both conventional ferroelectrics and liquid crystal ferroelectrics. After establishing the existence of the ferroelectric switching effect in SLG, we demonstrate potential device application in two‐terminal devices (Figure 6a). We demonstrate a bipolar polarization switching effect (Figure 7c,f). We attribute the switching behavior to a ferroelectric tunnel junction (FTJ) with diode‐like electroresistance behavior.^[^ ^84^, ^89^, ^97^ ^]^ The arrows in Figure 7c represent the switching from high resistance state (HRS) to low resistance state (LRS). The corresponding dielectric response of the sandwiched SLG was measured with respect to temperature at a wide frequency range from 0.1 MHz up to 10 MHz (Figure S17, Supporting Information).

## 3 Conclusion

We show the existence of polar 2D metallic van der Waals structures of gallenene in their natural form under a liquid confinement approach. Our results provide details on the spontaneous polarization of a rare polar metallic 2D material with nonlinear optical properties. We show that liquid gallium encapsulated between graphene layers resulted in an AB stacked configuration of single‐indexed gallium crystals with a100 orientation. In this configuration, the AB‐stacked gallenene (a100) crystals exhibit spontaneous ferroelectric polarization. The ferroelectric polarization measurement agrees with ferroelectric liquid crystals^[^ ^46^, ^98^ ^]^ and unconventional ferroelectric materials.^[^ ^99^ ^]^ Our experimental findings are supported by DFT calculations, which revealed the piezoelectric attributes of AB layer‐stacked gallenene with a piezoelectric tensor unit of 10^−10^ *C*/*m*. The value agrees with the spontaneous polarization of 2D single‐element bismuth monolayer (*Ps*  =  0.41  ×  10^−10^ *C*/*m*)^[^ ^3^ ^]^ and higher than that of 4‐layer van der Waals heterostructure.^[^ ^100^ ^]^ The nonlinear optical response characterized using SHG further supports the non‐centrosymmetric polar behavior of gallenene crystals. The structural sensitivity of the multilayer gallenene structures is confirmed through external perturbations, including thermal heating, excitation polarization, angular rotation, and electric field. We demonstrate a practical application as a resistive switching device with a bipolar current‐voltage loop indicating low and high resistance states. Our results are likely to motivate studies on the metallic ferroelectric, piezoelectric, and topological phenomena of van der Waals 2D gallenene.

## 4 Experimental Section

### Fabrication of SLG Sandwich Devices—Glass Preparation

For SLG imaging preparation, 18 mm x 18 mm x 500 µm and 12 mm x 12 mm x 120 µm transparent ITO coated glass slides were first cleaned in isopropanol‐solution and then rinsed in deionized (DI) water. After drying at room temperature, a 15 min UV‐ozone treatment was applied to the ITO coating side of the glass electrode. Then, homeotropic alignment material–polyimide (PI) (Nissan SE‐1211) was spin‐coated (10 s at 500 RPM, 45 s at 3000 RPM) on the glass electrode. The polymer coating on the ITO glass was cured in two steps, at 120 °C for 10 min and 180 °C for 90 min. The thickness of the polyimide coating was ≈100 nm. Then, the coated glass slides were rubbed with a velvet rubbing cloth. The rubbing introduced grooves on the glass surfaces, providing a preferred flow path for SLG. The PI‐coated glasses were cut by hand using a diamond‐tip glass‐cutting pen to encapsulate SLG. The glasses were cut to the dimensions of the microscope setups (9 mm x 18 mm x 500 µm and 9 mm x 12 mm x 120 µm). The device's thickness was defined using silica beads with 17 µm diameters. To secure the glass beads, UV glue (Norland Optical Adhesive 61) was applied and cured using a 365 nm UV light source for 15 min to bind the two electrically conducting glasses together completely.

### Fabrication of SLG Sandwich Devices—SLG Sandwich Cell

Gallium (99.99999% purity) was purchased from Sigma Aldrich. The SLG was obtained by heating solid gallium above its melting point (29.8 °C). A droplet of SLG was on a 9 mm x 18 mm x 500 µm or 9 mm x 12 mm x 120 µm piece to prepare the devices. Once the SLG droplets were confined between the two ITO glasses that were rubbed or unrubbed (smooth) by shear flow, the cell was placed under UV light to cure the UV‐sensitive glue spacer with glass beads corresponding to the thickness. Sandwich devices were prepared with three different arrangements of lined and unlined glass structures, including 1) both top and bottom glass were unlined, 2) both top and bottom glass were lined, 3) top glass was unlined, and the bottom glass was unlined. Samples were initially prepared with lined glass on both the top and bottom for SHG imaging (2); however, the lines interfered with the SHG measurements. Therefore, unlined samples were prepared for imaging and samples that had a lined bottom glass and an unlined top glass (3).

### Fabrication of SLG Sandwich Devices—Electric Lead Connection

Two wire electrodes were connected to each conducting side of the ITO glasses using copper wires through silver conductive epoxy adhesive (MG Chemicals 8331‐14G). The silver paste was cured for 30 min to allow secure electrical connections between the copper wire and the electrically conducting glass. After SLG sandwich devices were prepared, they were heat‐treated. The specific type of SLG sandwich device used for each experiment is included in Table S1 (Supporting Information).

### Fabrication of SLG Sandwich Devices—Thermal Annealing

The prepared sandwich cells were thermally annealed in a Linkam (THMS600). A temperature‐controlled stage mounted on a ZEISS Axio Imager.Z2 enabled in situ thermal annealing of the cells at heating and cooling rates of 5 °C/min. The devices were annealed from room temperature up to 160 °C and stabilized for 30 min before cooling down to room temperature at the same rate (5 °C/min). Precise cooling control of the sample was achieved via liquid nitrogen cooling through the LNP96 cooling option.

### Fabrication of SLG Sandwich Devices—Graphene Encapsulation

A commercially available monolayer graphene‐coated SiN membrane (PELCO Single Layer Graphene on a perforated Silicon Nitride, 2.5 µm holes, 0.5 × 0.5 mm window) was used to encapsulate SLG for atomic resolution imaging via TEM. Lab synthesized monolayer graphene was used for the top graphene encapsulation layer. Using TEM allowed us to probe the atomic structure of SLG on an atomic scale. The graphene monolayer serves as a buffer layer, which provides a hexagonal template to promote and induce long‐range order of gallenene (100) stacking.

### Material Characterization of SLG—Optical Measurements

Droplets of Liquid Ga were placed on glass slides. A polarized light microscope (ZEISS Axio Imager Z2, Carl Zeiss Inc., Oberkochen, Germany) was used in both reflection and transmission modes to observe freestanding gallenene and filaments stabilized at three different scales (Figure S1, Supporting Information). Phase differences were observed with polarized light and by rotating the stage 45 degrees.

### Material Characterization of SLG—Scanning Electron Microscopy

Zeiss Ultra 500 Gemini SEM (Carl Zeiss Inc., Oberkochen, Germany) was used to characterize the microstructure and morphology of Ga films. A liquid gallium droplet was trapped and solidified between the glass slide and polydimethylsiloxane (PDMS) surfaces. The film (flattened droplet) was frozen and fractured after immersion in liquid nitrogen for 2 min. The fractured film was examined in SEM using a 1.5 kV electron beam energy at −20 °C to avoid structural damage. The sample was maintained at −20 °C throughout the imaging using a built‐in cooling system.

### Material Characterization of SLG—Atomic Force Microscopy (AFM) Analysis

AFM of transparent lamellar films and helical filaments was performed using Bruker JPK Nanowizard 4 in contact mode. An Arrow‐Contr‐10 cantilever was used and calibrated at 14 kHz resonance frequency and 0.2 Nm^−1^ force constant.

### Material Characterization of SLG—Scanning Transmission Electron Microscopy Characterization

STEM experiments were performed using a Nion UltraSTEM100 operated at a 60 kV accelerating voltage in ultra‐high vacuum. STEM images were acquired using a HAADF detector with collection angles of 80–300. The prepared graphene‐encapsulated liquid metal sandwich device was heated at 150 °C prior to imaging in STEM. EELS measurements were performed with a Gatan PEELS 666 spectrometer retrofitted with an Andor iXon 897 electron‐multiplying charge‐coupled device camera. The following parameters were used for the EELS experiments: The energy dispersion of 0.5 eV, the beam current of 30 pA, and the EELS collection half angle of 35 mrad.

HAADF image simulations were performed using QSTEM software with the following parameters used in the experiments: Chromatic aberration coefficient of 1 mm, spherical aberration coefficient of 1 µm, and energy spread of 0.48 eV. In the simulations, the angular ranges of the HAADF detectors were set to the experimental range of 80–300 mrad.

### Material Characterization of SLG—Nuclear Magnetic Resonance

NMR samples were prepared by filling supercooled liquid gallium into commercially available microcapillaries, Hilgenberg GmbH (200 and 300 µm in diameter). Then, 20 to 30 pieces of the liquid gallium filled capillaries were inserted into the NMR tube in an upright position, covering at least a 2 cm sample window for the NMR spectroscopy. All NMR measurements were carried out on a Bruker Avance III 400 MHz spectrometer in a magnetic field of 9.4 T. The experiments use a Bruker double resonance BBO 5 mm multinuclear probe head with automatic tuning and matching.

The Knight shift of the NMR signal was obtained for the isotope of ^71^Ga at the resonance frequency of 122 MHz. To detect the NMR signal, a 90‐degree selective pulse for the nucleus was set at 9 µs (RF = 50 kHz) using the spin echo technique. The spectrum was obtained by accumulating 512 scans with a relaxation delay of 0.2 s for a better signal‐to‐noise. The ^71^Ga shift was measured relative to the signals of 1 M Ga_2_(SO_4_)_3_.

### Nonlinear Optical Measurements of Supercooled Liquid Ga—Beam Excitation Angle Shifts

The linear polarization of the laser was manipulated by a λ/2 waveplate implemented into the beam path. Rotating it by an angle of Θ leads to a linear polarization shift of 2Θ. The susceptibility of the liquid gallium sample was investigated for full cycles of polarization rotation in steps of ΔΘ = 5°, recording a new image and acquiring new spectra for each step to observe induced changes. For parts of the experiment, a Glan–Thompson prism was implemented before the detector to differentiate between s‐ and p‐polarized signals. For this, the Glan–Thompson analyzer was positioned at two different rotations for each excitation electric field rotation, i.e., parallel (ΔΘ = 0°) and perpendicular (ΔΘ = 90°) to it.

### Nonlinear Optical Measurements of Supercooled Liquid Ga—Temperature Variation

The temperature in situ manipulation was done with a Peltier module (CP074965‐238P, CUI Devices). Copper wires were routed into the parabolic mirror along the sample holder stage to minimize optical signal loss. During the heating procedure, the voltage applied to the Peltier module was increased in steps of ΔU = 0.05 V. Exact current values in mA were recorded for precise reproducibility (Figure S18, Supporting Information). After the applied voltage was increased, the sample was left to adjust to the new temperature for 3 min before the image and spectra were recorded. A temperature curve of the liquid gallium sample attached to the Peltier module was recorded after the measurement (Figure S18, Supporting Information).

### Nonlinear Optical Measurements of Supercooled Liquid Ga—Voltage Application

Copper wires were attached to the coated surfaces of the top and bottom glass to create a closed circuit. A voltage source (PL154 15V‐4A PSU, Thurlby Thandar Instruments) applied voltage to the liquid gallium sample in steps of ΔU = 1, 2, 10, or 20 V. There was a 1‐min wait after the voltage was changed. The starting voltage varied between 0 V, +10 V, and ‐10 V for different measurements.

### Ferroelectric Characterization of SLG—Piezoresponse Force Microscopy (PFM)

Piezoresponse force microscopy (PFM) measurements were conducted using a Cypher ES atomic force microscope (Asylum Research, Oxford Instruments) operating in dual AC resonance tracking mode (DART‐PFM). All PFM measurements were performed using conductive Ti/Ir‐coated silicon probes (ASYELEC.01‐R2, Oxford Instruments), with a nominal spring constant of 2.8 N m^−1^ and a resonance frequency of 75 kHz. The contact resonance frequency during DART‐PFM measurements was typically ≈300 kHz. The GetReal function in the Asylum software was used to calibrate both the spring constant and inverse optical lever sensitivity before all measurements. The local piezoresponse hysteresis loops were acquired using DART‐SS (switching spectroscopy)‐PFM mode by applying a DC bias sweep from ‐4 to +4 V superimposed on a 1 V AC excitation at the contact resonance frequency. Each measurement consisted of 8 cycles, allowing the collection of both the “on” (applied) and “off” (remnant) loops.

### Nonlinear Optical Measurements of Supercooled Liquid Ga—Dielectric Measurements

Dielectric measurements over a frequency range of 0.1–10 MHz were performed using KEYSIGHT ENA Network Analyzer (5 Hz–3 GHz). The glass substrates coated with low sheet resistance ITO electrodes (20 Ω per square) were used to make cells. The experimental setup was calibrated by prior measurement of the capacitance of the empty cell. The measurement was carried out under the application of a weak field with 0.1 Vµm^−1^.

## Conflict of Interest

The authors declare no conflict of interest.

## Author Contributions

M.Y., A.K.S., T.P., F.S., and K.E. contributed equally to this work. M.Y. and A.K.S. conceived the project. M.Y. and A.K.S. fabricated devices and conducted electrical measurements. T.P. and F.S. performed S.H.G. measurements. K.E., M.P., J.D., and J.K. performed STEM analysis. T.G. and E.D. conducted D.F.T. calculations. J.Y., J.C.T., and W.K. performed P.F.M. experiments. M.Y., A.K.S., T.P., F.S., M.R., M.P., J.D., K.E., T.G., J.Y., and W.K. analysed and interpreted the data. M.Y., A.K.S., J.K., D.Z., and M.S. supervised the research. M.Y., A.K.S., T.P., F.S., and K.E. wrote the manuscript with input from M.R., M.P., J.D., T.G., J.Y., J.C.T., W.K., P.A.v.A., A.J.M., E.D., J.K., D.Z., and M.S. All authors reviewed and commented on the manuscript.

## Supporting information



Supporting Information

Supplemental Video 1

## Data Availability

The data that support the findings of this study are available in the supplementary material of this article.

## References

[1] P. W. Anderson , E. I. Blount , Phys. Rev. Lett. 1965, 14, 217.

[2] P. Sharma , F.‐X. Xiang , D.‐F. Shao , D. Zhang , E. Y. Tsymbal , A. R. Hamilton , J. Seidel , Sci. Adv. 2019, 5, aax5080. 10.1126/sciadv.aax5080 PMC6611688 31281902

[3] J. Gou , H. Bai , X. Zhang , Y. L. Huang , S. Duan , A. Ariando , S. A. Yang , L. Chen , Y. Lu , A. T. S. Wee , Nature 2023, 617, 67. 37020017 10.1038/s41586-023-05848-5 PMC10156600

[4] R. Niu , Z. Li , X. Han , Z. Qu , D. Ding , Z. Wang , Q. Liu , T. Liu , C. Han , K. Watanabe , T. Taniguchi , M. Wu , Q. Ren , X. Wang , J. Hong , J. Mao , Z. Han , K. Liu , Z. Gan , J. Lu , Nat. Commun. 2022, 13, 6241. 36271005 10.1038/s41467-022-34104-z PMC9587233

[5] M. Vizner Stern , Y. Waschitz , W. Cao , I. Nevo , K. Watanabe , T. Taniguchi , E. Sela , M. Urbakh , O. Hod , M. Ben Shalom , Science 2021, 372, 1462. 10.1126/science.abe8177 34112727

[6] K. Yasuda , X. Wang , K. Watanabe , T. Taniguchi , P. Jarillo‐Herrero , Science 2021, 372, 1458. 10.1126/science.abd3230 34045323

[7] F. Sui , M. Jin , Y. Zhang , R. Qi , Y.‐N. Wu , R. Huang , F. Yue , J. Chu , Nat. Commun. 2023, 14, 36. 36596789 10.1038/s41467-022-35490-0 PMC9810696

[8] A. Weston , E. G. Castanon , V. Enaldiev , F. Ferreira , S. Bhattacharjee , S. Xu , H. Corte‐León , Z. Wu , N. Clark , A. Summerfield , T. Hashimoto , Y. Gao , W. Wang , M. Hamer , H. Read , L. Fumagalli , A. V. Kretinin , S. J. Haigh , O. Kazakova , A. K. Geim , V. I. Fal'ko , R. Gorbachev , Nat. Nanotechnol. 2022, 17, 390. 35210566 10.1038/s41565-022-01072-w PMC9018412

[9] X. Wang , K. Yasuda , Y. Zhang , S. Liu , K. Watanabe , T. Taniguchi , J. Hone , L. Fu , P. Jarillo‐Herrero , Nat. Nanotechnol. 2022, 17, 367. 35039684 10.1038/s41565-021-01059-z

[10] N. Briggs , B. Bersch , Y. Wang , J. Jiang , R. J. Koch , N. Nayir , K. Wang , M. Kolmer , W. Ko , A. De La Fuente Duran , S. Subramanian , C. Dong , J. Shallenberger , M. Fu , Q. Zou , Y.‐W. Chuang , Z. Gai , A.‐P. Li , A. Bostwick , C. Jozwiak , C.‐Z. Chang , E. Rotenberg , J. Zhu , A. C. T. van Duin , V. Crespi , J. A. Robinson , Nat. Mater. 2020, 19, 637. 32157191 10.1038/s41563-020-0631-x

[11] M. A. Steves , Y. Wang , N. Briggs , T. Zhao , H. El‐Sherif , B. M. Bersch , S. Subramanian , C. Dong , T. Bowen , A. D. L. Fuente Duran , K. Nisi , M. Lassaunière , U. Wurstbauer , N. D. Bassim , J. Fonseca , J. T. Robinson , V. H. Crespi , J. Robinson , K. L. Knappenberger Jr , Nano Lett. 2020, 20, 8312. 33079555 10.1021/acs.nanolett.0c03481

[12] W. He , M. T. Wetherington , K. A. Ulman , J. L. Gray , J. A. Robinson , S. Y. Quek , J. Phys. Chem. Lett. 2022, 13, 4015. 35485838 10.1021/acs.jpclett.2c00719

[13] C. Li , Y.‐F. Zhao , A. Vera , O. Lesser , H. Yi , S. Kumari , Z. Yan , C. Dong , T. Bowen , K. Wang , H. Wang , J. L. Thompson , K. Watanabe , T. Taniguchi , D. R. Hickey , Y. Oreg , J. A. Robinson , C.‐Z. Chang , J. Zhu , Nat. Mater. 2023, 22, 570. 36781950 10.1038/s41563-023-01478-4

[14] V. Kochat , A. Samanta , Y. Zhang , S. Bhowmick , P. Manimunda , S. A. S. Asif , A. S. Stender , R. Vajtai , A. K. Singh , C. S. Tiwary , P. M. Ajayan , Sci. Adv. 2018, 4, 1701373. 10.1126/sciadv.1701373 PMC5844710 29536039

[15] S. Lambie , K. G. Steenbergen , N. Gaston , Nanoscale Adv 2021, 3, 499. 36131742 10.1039/d0na00737d PMC9418766

[16] D. Z. Metin , L. Hammerschmidt , N. Gaston , Phys. Chem. Chem. Phys. 2018, 20, 27668. 30375598 10.1039/c8cp05280h

[17] S. Wundrack , D. Momeni , W. Dempwolf , N. Schmidt , K. Pierz , L. Michaliszyn , H. Spende , A. Schmidt , H. W. Schumacher , R. Stosch , A. Bakin , Phys. Rev. Mater. 2021, 5, 024006.

[18] H. El‐Sherif , N. Briggs , B. Bersch , M. Pan , M. Hamidinejad , S. Rajabpour , T. Filleter , K. W. Kim , J. Robinson , N. D. Bassim , ACS Appl. Mater. Interfaces 2021, 13, 55428. 34780159 10.1021/acsami.1c14091

[19] M.‐L. Tao , Y.‐B. Tu , K. Sun , J. Ye , S.‐J. Hao , H.‐F. Xiao , Y.‐L. Wang , Z.‐B. Xie , J.‐Z. Wang , Surf. Sci. 2017, 663, 31.

[20] L. V. Bondarenko , A. Y. Tupchaya , Y. E. Vekovshinin , D. V. Gruznev , A. N. Mihalyuk , D. V. Denisov , A. V. Matetskiy , D. A. Olyanich , T. V. Utas , V. S. Zhdanov , A. V. Zotov , A. A. Saranin , Mol. Syst. Des. Eng. 2023, 8, 604.

[21] H.‐M. Zhang , Y. Sun , W. Li , J.‐P. Peng , C.‐L. Song , Y. Xing , Q. Zhang , J. Guan , Z. Li , Y. Zhao , S. Ji , L. Wang , K. He , X. Chen , L. Gu , L. Ling , M. Tian , L. Li , X. C. Xie , J. Liu , H. Yang , Q.‐K. Xue , J. Wang , X. Ma , Phys. Rev. Lett. 2015, 114, 107003. 25815961 10.1103/PhysRevLett.114.107003

[22] E. Durgun , S. Dag , S. Ciraci , Phys. Rev. B 2004, 70, 155305.

[23] M. Yunusa , A. Adaka , A. Aghakhani , H. Shahsavan , Y. Guo , Y. Alapan , A. Jákli , M. Sitti , Adv. Mater. 2021, 33, 2104807. 34337803 10.1002/adma.202104807 PMC11468993

[24] S. Yu , M. Kaviany , J. Chem. Phys. 2014, 140, 064303. 24527911 10.1063/1.4865105

[25] T. Daeneke , K. Khoshmanesh , N. Mahmood , I. A. de Castro , D. Esrafilzadeh , S. J. Barrow , M. D. Dickey , K. Kalantar‐zadeh , Chem. Soc. Rev. 2018, 47, 4073. 29611563 10.1039/c7cs00043j

[26] E. J. Markvicka , M. D. Bartlett , X. Huang , C. Majidi , Nature Mater 2018, 17, 618. 29784995 10.1038/s41563-018-0084-7

[27] M. D. Dickey , Adv. Mater. 2017, 29, 1606425.

[28] J. Xu , H. Guo , H. Ding , Q. Wang , Z. Tang , Z. Li , G. Sun , ACS Appl. Mater. Interfaces 2021, 13, 7443. 33528998 10.1021/acsami.0c20549

[29] W. Xie , F.‐M. Allioux , J. Z. Ou , E. Miyako , S.‐Y. Tang , K. Kalantar‐Zadeh , Trends Biotechnol. 2021, 39, 624. 33199046 10.1016/j.tibtech.2020.10.005

[30] R. Li , G. Sun , L. Xu , J. Chem. Phys. 2016, 145, 054506. 27497564 10.1063/1.4959891

[31] S.‐Y. Tang , D. R. G. Mitchell , Q. Zhao , D. Yuan , G. Yun , Y. Zhang , R. Qiao , Y. Lin , M. D. Dickey , W. Li , Matter 2019, 1, 192.

[32] R. Li , L. Wang , L. Li , T. Yu , H. Zhao , K. W. Chapman , Y. Wang , M. L. Rivers , P. J. Chupas , H. Mao , H. Liu , Sci. Rep. 2017, 7, 5666. 28720773 10.1038/s41598-017-05985-8 PMC5515953

[33] W. Kong , Z. Wang , M. Wang , K. C. Manning , A. Uppal , M. D. Green , R. Y. Wang , K. Rykaczewski , Adv. Mater. 2019, 31, 1904309. 10.1002/adma.201904309 31523854

[34] K. H. Matlack , J.‐Y. Kim , L. J. Jacobs , J. Qu , J Nondestruct Eval 2014, 34, 273.

[35] V. K. Valev , Langmuir 2012, 28, 15454. 22889193 10.1021/la302485c

[36] C. J. Boyle , M. Plotczyk , S. F. Villalta , S. Patel , S. Hettiaratchy , S. D. Masouros , M. A. Masen , C. A. Higgins , Sci. Adv 2019, 5, aay0244. 10.1126/sciadv.aay0244 PMC6785259 31633031

[37] A. K. Schulz , M. Plotczyk , S. Sordilla , D. C. A. Gaboriau , M. Boyle , K. Singal , J. S. Reidenberg , D. L. Hu , C. A. Higgins , Commun. Biol. 2025, 8, 17. 39779804 10.1038/s42003-024-07386-w PMC11711191

[38] C.‐C. Zhang , J.‐Y. Zhang , J.‐R. Feng , S.‐T. Liu , S.‐J. Ding , L. Ma , Q.‐Q. Wang , Nanoscale 2024, 16, 5960. 38446099 10.1039/d3nr06675d

[39] H. W. K. Tom , G. D. Aumiller , Phys. Rev. B 1986, 33, 8818. 10.1103/physrevb.33.8818 9938298

[40] S.‐P. Guo , X. Cheng , Z.‐D. Sun , Y. Chi , B.‐W. Liu , X.‐M. Jiang , S.‐F. Li , H.‐G. Xue , S. Deng , V. Duppel , J. Köhler , G.‐C. Guo , Angew. Chem., Int. Ed. 2019, 58, 8087. 10.1002/anie.201902839 31002447

[41] E. Gürdal , A. Horneber , N. Shaqqura , A. J. Meixner , D. P. Kern , D. Zhang , M. Fleischer , J. Chem. Phys. 2020, 152, 104711. 32171201 10.1063/1.5139893

[42] J. Wang , J. Butet , G. D. Bernasconi , A.‐L. Baudrion , G. Lévêque , A. Horrer , A. Horneber , O. J. F. Martin , A. J. Meixner , M. Fleischer , P.‐M. Adam , D. Zhang , Nanoscale 2019, 11, 23475. 31799534 10.1039/c9nr07644a

[43] L. M. Malard , T. V. Alencar , A. P. M. Barboza , K. F. Mak , A. M. de Paula , Phys. Rev. B 2013, 87, 201401.

[44] S. Psilodimitrakopoulos , L. Mouchliadis , I. Paradisanos , A. Lemonis , G. Kioseoglou , E. Stratakis , Light Sci Appl 2018, 7, 18005. 30839517 10.1038/lsa.2018.5 PMC6060071

[45] L. Pan , P. Miao , A. Horneber , A. J. Meixner , P.‐M. Adam , D. Zhang , ACS Appl. Nano Mater 2023, 6, 6467.

[46] H. Nishikawa , K. Shiroshita , H. Higuchi , Y. Okumura , Y. Haseba , S. Yamamoto , K. Sago , H. Kikuchi , Adv. Mater. 2017, 29, 1702354. 10.1002/adma.201702354 29023971

[47] X. Wang , Z. Zeng , X. Zhuang , F. Wackenhut , A. Pan , A. J. Meixner , Opt. Lett. 2017, 42, 2623. 28957300 10.1364/OL.42.002623

[48] E. Tsai , J. M. Richardson , E. Korblova , M. Nakata , D. Chen , Y. Shen , R. Shao , N. A. Clark , D. M. Walba , Angew. Chem., Int. Ed. 2013, 52, 5254. 10.1002/anie.201209453 23606222

[49] D. Chen , J. E. Maclennan , R. Shao , D. K. Yoon , H. Wang , E. Korblova , D. M. Walba , M. A. Glaser , N. A. Clark , J. Am. Chem. Soc. 2011, 133, 12656. 21692442 10.1021/ja203522x

[50] L. E. Hough , H. T. Jung , D. Krüerke , M. S. Heberling , M. Nakata , C. D. Jones , D. Chen , D. R. Link , J. Zasadzinski , G. Heppke , J. P. Rabe , W. Stocker , E. Körblova , D. M. Walba , M. A. Glaser , N. A. Clark , Science 2009, 325, 456. 19628864 10.1126/science.1170027

[51] B. Mendiburu , in Handbook of Visual Display Technology (Eds.: J. Chen , W. Cranton , M. Fihn ), Springer, Berlin, Heidelberg 2016, pp. 1–14.

[52] S.‐C. Jeng , S.‐J. Hwang , S.‐C. Jeng , S.‐J. Hwang , in High Performance Polymers – Polyimides Based – From Chemistry to Applications, IntechOpen, London Uk 2012, pp. 88–104.

[53] Z. Xu , C. Gao , Nat. Commun. 2011, 2, 571. 22146390 10.1038/ncomms1583 PMC3247827

[54] K. Elibol , T. Susi , C. Mangler , D. Eder , J. C. Meyer , J. Kotakoski , R. G. Hobbs , P. A. van Aken , B. C. Bayer , npj 2D Mater. Appl. 2023, 7, 2. 38665487 10.1038/s41699-023-00364-6 PMC11041670

[55] K. Elibol , C. Mangler , D. D. O'Regan , K. Mustonen , D. Eder , J. C. Meyer , J. Kotakoski , R. G. Hobbs , T. Susi , B. C. Bayer , ACS Nano 2021, 15, 14373. 34410707 10.1021/acsnano.1c03535 PMC8482752

[56] O. Züger , U. Dürig , Ultramicroscopy 1992, 47, 520.

[57] E. V. Charnaya , D. Michel , C. Tien , Y. A. Kumzerov , D. Yaskov , J. Phys.: Condens. Matter 2003, 15, 5469.

[58] C. Tien , E. V. Charnaya , P. Sedykh , Y.u. A. Kumzerov , Phys. Solid State 2003, 45, 2352.

[59] E. Shabanova , E. V. Charnaya , K. Schaumburg , Y. A. Kumzerov , Phys. B 1997, 229, 268.

[60] C. Tien , E. V. Charnaya , W. Wang , Y. A. Kumzerov , D. Michel , Phys. Rev. B 2006, 74, 024116.

[61] M. Petrov , J. Bekaert , M. V. Milošević , 2D Mater. 2021, 8, 035056.

[62] X. Li , J. Qiu , H. Cui , X. Chen , J. Yu , K. Zheng , ACS Appl. Mater. Interfaces 2024, 16, 12731. 38421155 10.1021/acsami.3c14610

[63] L. Dong , J. Lou , V. B. Shenoy , ACS Nano 2017, 11, 8242. 28700210 10.1021/acsnano.7b03313

[64] (Eds.: F. S. Pavone , P. J. Campagnola ), in Second Harmonic Generation Imaging, CRC Press, Boca Raton 2013.

[65] D. A. Kleinman , Phys. Rev. 1962, 128, 1761.

[66] F. Simon , S. Clevers , V. Dupray , G. Coquerel , Chem. Eng. Technol. 2015, 38, 971.

[67] M. A. Lieb , A. J. Meixner , Opt. Express, OE 2001, 8, 458. 10.1364/oe.8.000458 19417842

[68] L.‐Q. Yang , X.‐M. Jiang , Y. Chen , B.‐W. Liu , G.‐C. Guo , Adv. Opt. Mater. 2024, 12, 2301897.

[69] R. G. dos Santos , L. J. Q. Maia , C. B. de Araújo , L. S. de Menezes , Chin. Opt. Lett. 2018, 16, 041902.

[70] M. Acosta , N. Novak , V. Rojas , S. Patel , R. Vaish , J. Koruza , G. A. Rossetti Jr., J. Rödel , Appl. Phys. Rev. 2017, 4, 041305.

[71] E. Kim , A. Steinbrück , M. T. Buscaglia , V. Buscaglia , T. Pertsch , R. Grange , ACS Nano 2013, 7, 5343. 23691915 10.1021/nn401198g

[72] S. A. Denev , T. T. A. Lummen , E. Barnes , A. Kumar , V. Gopalan , J. Am. Ceram. Soc. 2011, 94, 2699.

[73] A. R. Khan , B. Liu , L. Zhang , Y. Zhu , X. He , L. Zhang , T. Lü , Y. Lu , Adv. Opt. Mater. 2020, 8, 2000441.

[74] A. Abulikemu , Y. Kainuma , T. An , M. Hase , Opt. Lett. 2022, 47, 1693. 35363710 10.1364/OL.455437

[75] L. H. Xiong , X. D. Wang , Q. Yu , H. Zhang , F. Zhang , Y. Sun , Q. P. Cao , H. L. Xie , T. Q. Xiao , D. X. Zhang , C. Z. Wang , K. M. Ho , Y. Ren , J. Z. Jiang , Acta Mater. 2017, 128, 304.

[76] H. Kikuchi , H. Matsukizono , K. Iwamatsu , S. Endo , S. Anan , Y. Okumura , Adv. Sci. 2022, 9, 2202048. 10.1002/advs.202202048 PMC9475520 35869031

[77] N. Sebastián , R. J. Mandle , A. Petelin , A. Eremin , A. Mertelj , Liq. Cryst. 2021, 48, 2055.

[78] A. Murad , E. Baron , M. Feneberg , M. Baumann , M. Lehmann , A. Eremin , ACS Appl. Mater. Interfaces 2024, 16, 25025. 38709679 10.1021/acsami.4c01900 PMC11103660

[79] Y. Q. An , J. E. Rowe , D. B. Dougherty , J. U. Lee , A. C. Diebold , Phys. Rev. B 2014, 89, 115310.

[80] Y. Wang , J. Xiao , S. Yang , Y. Wang , X. Zhang , Opt. Mater. Express 2019, 9, 1136.

[81] J. Wissman , M. D. Dickey , C. Majidi , Adv. Sci. 2017, 4, 1700169. 10.1002/advs.201700169 PMC5737232 29270335

[82] K. Miyasato , S. Abe , H. Takezoe , A. Fukuda , E. Kuze , Jpn. J. Appl. Phys. 1983, 22, L661.

[83] C. J. Gibb , J. Hobbs , D. I. Nikolova , T. Raistrick , S. R. Berrow , A. Mertelj , N. Osterman , N. Sebastián , H. F. Gleeson , R. J. Mandle , Nat. Commun. 2024, 15, 5845. 38992039 10.1038/s41467-024-50230-2 PMC11239904

[84] F. Liu , L. You , K. L. Seyler , X. Li , P. Yu , J. Lin , X. Wang , J. Zhou , H. Wang , H. He , S. T. Pantelides , W. Zhou , P. Sharma , X. Xu , P. M. Ajayan , J. Wang , Z. Liu , Nat. Commun. 2016, 7, 12357. 27510418 10.1038/ncomms12357 PMC4987531

[85] W. F. Io , S.‐Y. Pang , L. W. Wong , Y. Zhao , R. Ding , J. Mao , Y. Zhao , F. Guo , S. Yuan , J. Zhao , J. Yi , J. Hao , Nat. Commun. 2023, 14, 7304. 37951934 10.1038/s41467-023-43097-2 PMC10640637

[86] A. Belianinov , Q. He , A. Dziaugys , P. Maksymovych , E. Eliseev , A. Borisevich , A. Morozovska , J. Banys , Y. Vysochanskii , S. V. Kalinin , Nano Lett. 2015, 15, 3808. 25932503 10.1021/acs.nanolett.5b00491

[87] L. Rogée , L. Wang , Y. Zhang , S. Cai , P. Wang , M. Chhowalla , W. Ji , S. P. Lau , Science 2022, 376, 973. 35617404 10.1126/science.abm5734

[88] P. Maksymovych , S. Jesse , P. Yu , R. Ramesh , A. P. Baddorf , S. V. Kalinin , Science 2009, 324, 1421. 19520954 10.1126/science.1171200

[89] Y. Jia , Q. Yang , Y.‐W. Fang , Y. Lu , M. Xie , J. Wei , J. Tian , L. Zhang , R. Yang , Nat. Commun. 2024, 15, 693. 38267445 10.1038/s41467-024-44927-7 PMC10808203

[90] Y. Zhou , D. Wu , Y. Zhu , Y. Cho , Q. He , X. Yang , K. Herrera , Z. Chu , Y. Han , M. C. Downer , H. Peng , K. Lai , Nano Lett. 2017, 17, 5508. 28841328 10.1021/acs.nanolett.7b02198

[91] J. Zhang , J. Zhang , Y. Qi , S. Gong , H. Xu , Z. Liu , R. Zhang , M. A. Sadi , D. Sychev , R. Zhao , H. Yang , Z. Wu , D. Cui , L. Wang , C. Ma , X. Wu , J. Gao , Y. P. Chen , X. Wang , Y. Jiang , Nat. Commun. 2024, 15, 7648. 39223121 10.1038/s41467-024-52062-6 PMC11368953

[92] M. T. Becker , P. Oldroyd , N. Strkalj , M. L. Müller , G. G. Malliaras , J. L. MacManus‐Driscoll , Appl. Phys. Lett. 2023, 122, 173701.

[93] R. A. Reddy , C. Zhu , R. Shao , E. Korblova , T. Gong , Y. Shen , E. Garcia , M. A. Glaser , J. E. Maclennan , D. M. Walba , N. A. Clark , Science 2011, 332, 72. 21454782 10.1126/science.1197248

[94] A. Akiyama , K. Jido , M. Kohri , T. Taniguchi , K. Kishikawa , Adv. Electron. Mater. 2020, 6, 2000201.

[95] J.‐H. So , H.‐J. Koo , M. D. Dickey , O. D. Velev , Adv. Funct. Mater. 2012, 22, 625.

[96] H.‐J. Koo , J.‐H. So , M. D. Dickey , O. D. Velev , Adv. Mater. 2011, 23, 3559. 21726000 10.1002/adma.201101257

[97] S. Sarkar , Z. Han , M. A. Ghani , N. Strkalj , J. H. Kim , Y. Wang , D. Jariwala , M. Chhowalla , Nano Lett. 2024, 24, 13232. 39382966 10.1021/acs.nanolett.4c03360 PMC11503766

[98] X. Chen , E. Korblova , D. Dong , X. Wei , R. Shao , L. Radzihovsky , M. A. Glaser , J. E. Maclennan , D. Bedrov , D. M. Walba , N. A. Clark , Proc. Natl. Acad. Sci. USA 2020, 117, 14021. 32522878 10.1073/pnas.2002290117 PMC7322023

[99] C. Zheng , L. Yu , L. Zhu , J. L. Collins , D. Kim , Y. Lou , C. Xu , M. Li , Z. Wei , Y. Zhang , M. T. Edmonds , S. Li , J. Seidel , Y. Zhu , J. Z. Liu , W.‐X. Tang , M. S. Fuhrer , Sci. Adv. 2018, 4, aar7720. 10.1126/sciadv.aar7720 PMC6044735 30027116

[100] X. Chen , X. Xuan , W. Guo , Z. Zhang , npj 2D Mater. Appl. 2025, 9, 10.

