# Supplementary material for: Nonlinear Optical Response in Layer‐Stacked Gallenene with Ferroelectric Polarization
Source: Adv Mater. 2025 Aug 21;37(44):e01058. doi: 10.1002/adma.202501058 (PMC12592908; doi:10.1002/adma.202501058)
Supplement: Supplementary file 1 — Supporting Information [file ADMA-37-e01058-s001.docx]

**Supplementary Information**

**Nonlinear Optical Response in Layer-Stacked Gallenene with Ferroelectric Polarization**

Muhammad Yunusa^1,†,*^, Andrew K. Schulz^2,†,*^, Tim Parker^3,†^, Felix Schneider^3,†^,Kenan Elibol^4,†^, Marius Predel^5^, Jana Dzíbelová^5^, Michel Rebmann^3^, Taylan Gorkan^6^, Jiahao Ye^7^, Jin-Chong Tan^7,^ Wenbin Kang^8^, Peter A. van Aken^4^, Alfred J. Meixner^3^, Engin Durgun^6^, Jani Kotakoski*^5^,* Dai Zhang^3, *^, Metin Sitti^1,9*^

^1^Physical Intelligence Department, Max Planck Institute for Intelligent Systems, 70569 Stuttgart, Germany;

^2^Max Planck Institute for Intelligent Systems, 70569 Stuttgart, Germany;

^3^Institute for Physical and Theoretical Chemistry, University of Tübingen and LISA+, 72076 Tübingen, Germany;

^4^Max Planck Institute for Solid State Research, 70569 Stuttgart, Germany;

^5^Faculty of Physics, University of Vienna, Boltzmanngasse 5, 1090 Vienna, Austria;

^6^UNAM-National Nanotechnology Research Center and Institute of Materials Science and Nanotechnology, Bilkent University, 06800 Ankara, Turkey;

^7^Multifunctional Materials and Composites (MMC) Laboratory, Department of Engineering Science, University of Oxford, Oxford, OX1 3PJ, UK;

^[8](https://www.cityu.edu.hk/mne/people/academic-staff/prof-kang-wenbin)^[[Department of Mechanical Engineering](https://www.cityu.edu.hk/mne/people/academic-staff/prof-kang-wenbin)](https://scholars.cityu.edu.hk/en/organisations/department-of-mechanical-engineering)[, City University of Hong Kong](https://www.cityu.edu.hk/mne/people/academic-staff/prof-kang-wenbin), 38 Tat Chee Avenue, Kowloon, Hong Kong, 999077, China;

^9^School of Medicine and College of Engineering, Koç University, 34450 Istanbul, Turkey

†These authors contributed equally to this work.

*Corresponding authors.

**Parabolic Mirror for Second Harmonic Generation (SHG) Imaging and Polarizer Calibration**

A home-built parabolic mirror microscope acquired SHG spectra and recorded SHG distribution images. The custom-built setup has been shown to work for a variety of materials.^[1–7]^ Exactly this home-built parabolic mirror microscope was previously used by our group to successfully characterize the second harmonic generation (SHG) of a wide range of materials, such as NbOI_2_ flakes as ferroelectric semiconductor^[1]^, LiNbO_3_ crystals^[2]^, MoSe_2_ triangular flakes^[3]^, WS_2_ triangular flakes^[4]^, Au and Al single nanodiscs and Au-Al nanodisc heterostructures^[5]^, ZnIn_2_S_4_ hexagonal monolayers and few-layer structures^[6]^, and Nb_3_SeI_7_ hexagonal monolayers and few-layer structures^[7]^. The studies on these materials have undergone extensive peer-review processes prior to publication, which makes us confident that the self-built parabolic mirror microscope is reliable and has been validated in various material studies.

The excitation laser is a pulsed laser with a wavelength of λ = 779 nm with a repetition frequency of 80 MHz (FemtoFiber pro, Toptica Photonics). Linear polarization in a Gaussian beam profile was ensured by implementing a Glan-Taylor polarizer in the beam path and adjusting it with a linear polarization filter (Fig. S11a). A long pass filter suppresses higher-order sidebands and only allows transmission of λ > 750 nm. Focusing and signal collection is done using a parabolic mirror with a numerical aperture of 0.9986 in air.^[8]^ After the signal is collected, the laser emission is blocked by either a bandpass filter that only allows transmission from λ = 380 nm to 400 nm, or a shortpass filter, only allowing transmission of $\lambda<680$ nm. Spectra were obtained with an Acton SP2500 spectrometer (Princeton Instruments) coupled to a Pixis 100 charge-coupled device (Princeton Instruments) using a 600 grooves/mm grating. For imaging the sample is scanned through the stationary focus using a high-precision nano-positioning stage (Physik Instrumente (PI)). At the same time, an avalanche photodiode (Count, Laser Components) records the intensity per pixel. The sample was scanned from the bottom to the top sandwiched glass (Fig. S19). After the supercooled liquid gallium (SLG) was found in the z-stack scan, spots were analyzed for their SHG signal (Fig. S210a-c).

The correct focal plane was identified by taking reference measurements on all focal planes. The first focal plane is assigned to the upper phase-boundary between glass and air, where circular structures with no SHG signal could be observed. They were not sensitive to optical, electrical or thermal perturbations. The second focal plane is assigned to the phase-boundary between glass and SLG, where strand-shaped structures with SHG signal could be observed, which were sensitive to optical, electrical and thermal perturbations. The background besides the structures showed no SHG. Since the optical signal includes SLG photoluminescence depending on the local structure, the photoluminescence (Fig. S20c) is superimposed with the SHG signal.^[9,10]^ Background correction is applied to compare corrected SHG signal intensity between multiple spots of interest (Fig. S20d).

**SHG validation with reference non-linear materials**

We conducted supplemental SHG experiments on two reference materials, namely β-BaB_2_O_4_ (beta-barium borate, BBO) as commonly accepted SHG reference material in the SHG-microscopy research field, and ferroelectric BaTiO_3_.

As shown in Figure 3d-e, both materials show strong SHG signals. Notably, the sensitivity of our microscope is too high to measure SHG for these three materials under the same excitation conditions that were used for the SLG experiments, as SHG intensity led to oversaturation of the CCD detector. Therefore, the laser power was reduced from 8 mW (SLG) to 5 mW (β-BaB_2_O_4_), and 1 mW (BaTiO_3_), and at the same time the acquisition time was reduced from 30 s (SLG) to 0.1 s (β-BaB_2_O_4_, and BaTiO_3_). We have performed beam electric field rotation experiments identical to those for the SLG sample included in the manuscript for both materials, using no polarization analyzer in the detection path, see Figure S8a-b.

The experimental results observed on our home-built parabolic mirror microscope are consistent with published experimental results on BaTiO_3_^[11,12]^, and β-BaB_2_O_4_^[13,14]^ in regards to their polarization dependent SHG response. For BaTiO_3_ and β-BaB_2_O_4_ we observe two-fold polarization dependent SHG responses, see Figure S8a-b, which is based on their crystal structure. The orientation of the two-fold pattern changes depending on the crystal orientation with respect to the laser polarization direction, as was also observed and explained for the gallenene in the manuscript.

**Computational Details for DFT**

The density functional theory (DFT) calculations were performed using the VASP package.^[15]^ The exchange-correlation potential was treated with the Perdew−Burke−Ernzerhof functional^[16]^ with the projected augmented wave pseudopotentials.^[17]^ A plane-wave basis set with a kinetic energy cutoﬀ of 500 eV was employed, and Brillouin zone integration was conducted using a 24×24×1 k-point mesh following the Monkhorst−Pack method.^[18]^ A Gaussian smearing method with a width of 0.05 eV was applied.

**AB-type Stacking Layer Dependent Piezoelectric Tensors in Unit (10−10 C/m)**

**Bilayer gallenene**

$$x=\left( \begin{matrix} 0.48 & -1.20 & 0.21 \\ -1.20 & -0.54 & -0.11 \\ 0.21 & -0.11 & 0.09 \end{matrix} \right), y=\left( \begin{matrix} 1.31 & 0.38 & -0.11 \\ 0.38 & -0.31 & -0.06 \\ -0.11 & -0.06 & -0.84 \end{matrix} \right), z=\left( \begin{matrix} 0.004 & 0.001 & 0.001 \\ 0.001 & -0.005 & 0.001 \\ 0.001 & 0.001 & 0.001 \end{matrix} \right)$$

**4-layer gallenene**

$$x=\left( \begin{matrix} -0.03 & -0.17 & -0.10 \\ -0.17 & 0.65 & 0.58 \\ -0.10 & 0.58 & -0.28 \end{matrix} \right),y=\left( \begin{matrix} 0.17 & -0.31 & 0.47 \\ -0.31 & 0.05 & 0.31 \\ 0.47 & 0.31 & -0.03 \end{matrix} \right),z=\left( \begin{matrix} -0.004 & 0.001 & 0.001 \\ 0.001 & 0.001 & 0.001 \\ 0.001 & 0.001 & -0.001 \end{matrix} \right)$$

**6-layer gallenene**

$$x=\left( \begin{matrix} 3.94 & 0.13 & 1.02 \\ 0.13 & 5.17 & -0.68 \\ 1.02 & -0.68 & 1.51 \end{matrix} \right),y=\left( \begin{matrix} -10.53 & -0.28 & -0.22 \\ -0.28 & -11.51 & 3.24 \\ -0.22 & 3.24 & -6.03 \end{matrix} \right), z=\left( \begin{matrix} 0.013 & -0.015 & -0.045 \\ -0.015 & -0.048 & 0.045 \\ -0.045 & 0.045 & -0.004 \end{matrix} \right)$$

**12-layer gallenene**

$$x=\left( \begin{matrix} 6.22 & -5.80 & -4.69 \\ -5.80 & 9.82 & 1.50 \\ -4.69 & 1.50 & 4.39 \end{matrix} \right), y=\left( \begin{matrix} -6.50 & 6.09 & 2.73 \\ 6.09 & -0.07 & 0.51 \\ 2.73 & 0.51 & -9.57 \end{matrix} \right), z=\left( \begin{matrix} -0.018 & 0.051 & -0.292 \\ 0.051 & 0.040 & 0.166 \\ -0.292 & 0.166 & 0.037 \end{matrix} \right)$$

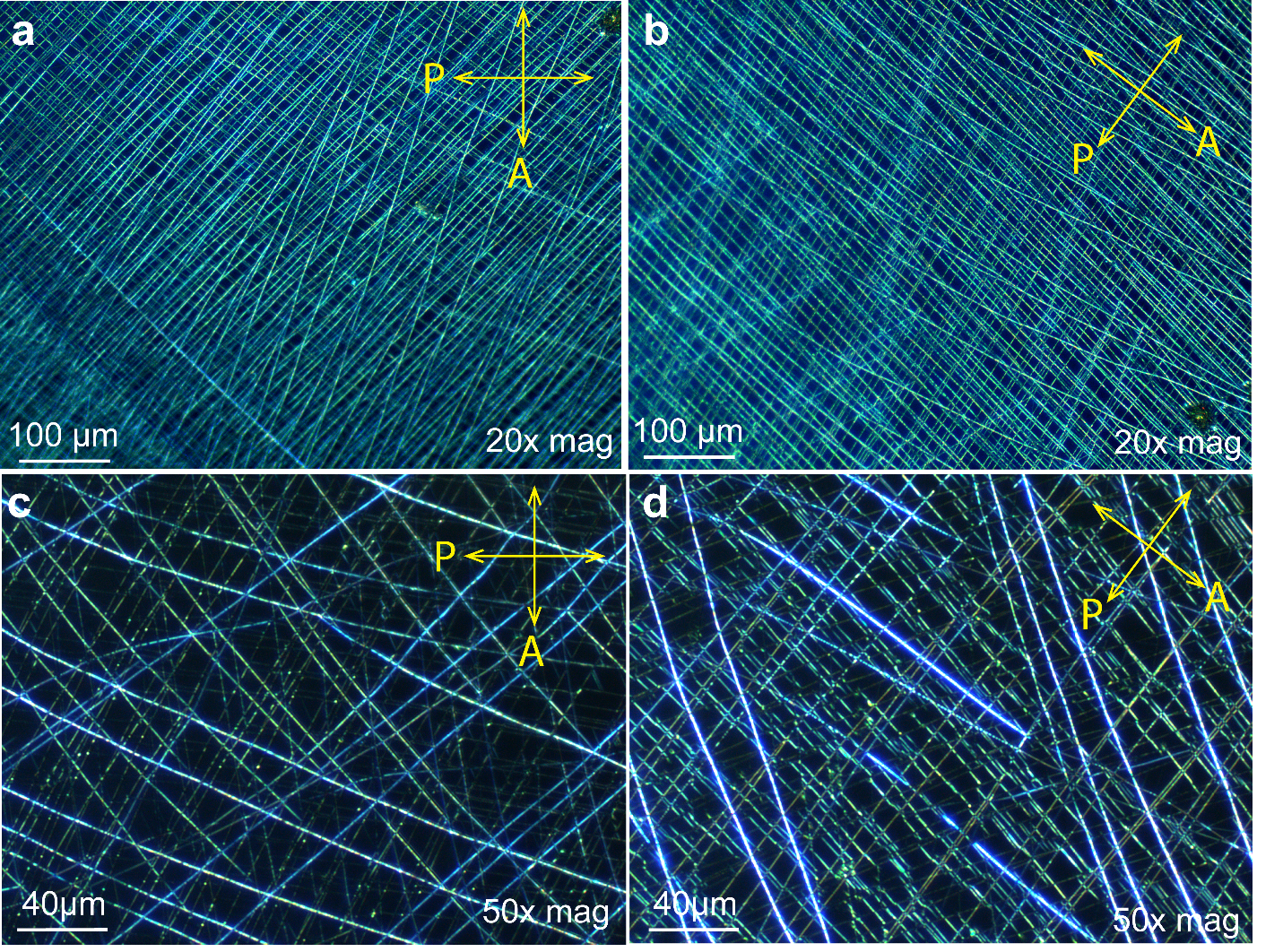


**Figure S1** | **Polarized light microscopy.** **a-b**, Supercooled liquid gallium (SLG) on PDMS showing thread-like texture of helical superstructures^[19]^ obtained at different magnifications in different sample rotations while fixed on the microscope stage shown in 20x magnification. **c-d**, A rotated sample shifting the primary axis shown in 50x magnification.

**
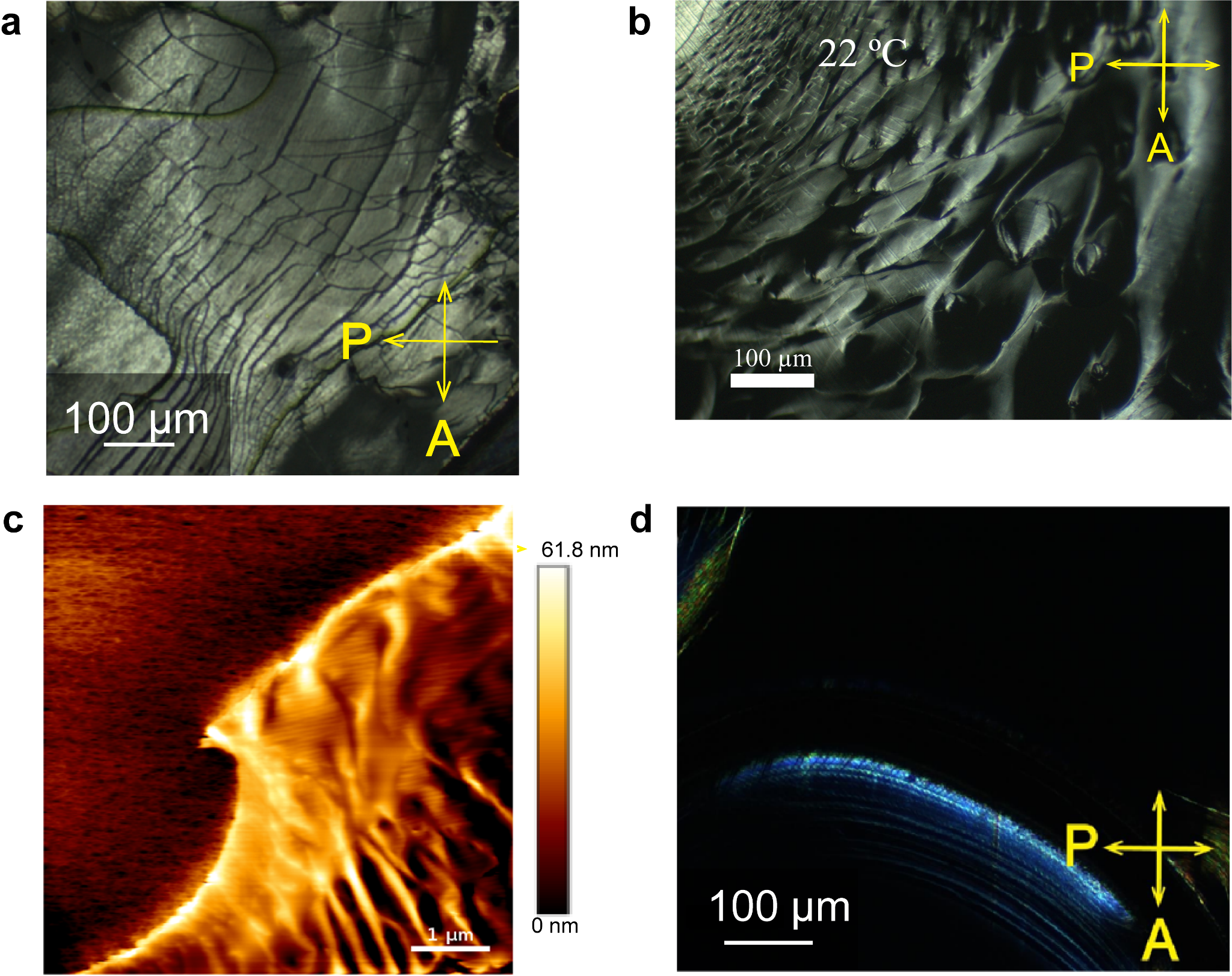
**

**Figure S2** | **Supercooled liquid gallium filaments.** **a**, Atomic force microscopy image of broken filaments on a glass surface. Layers are parallel to the glass surface, showing lamellar twist and folds. **b**, Linearly cross-polarized optical micrograph of filament growing from the confined SLG thin film at room temperature. **c**, Linearly cross-polarized optical micrograph of flat terraces of liquid exfoliated SLG. The yellow highlighted P and A, represent the polarizer and the analyzer. **d**, Polarized light microscopy of lamellar texture obtained from (SLG) showing layer twist on an acrylic surface.


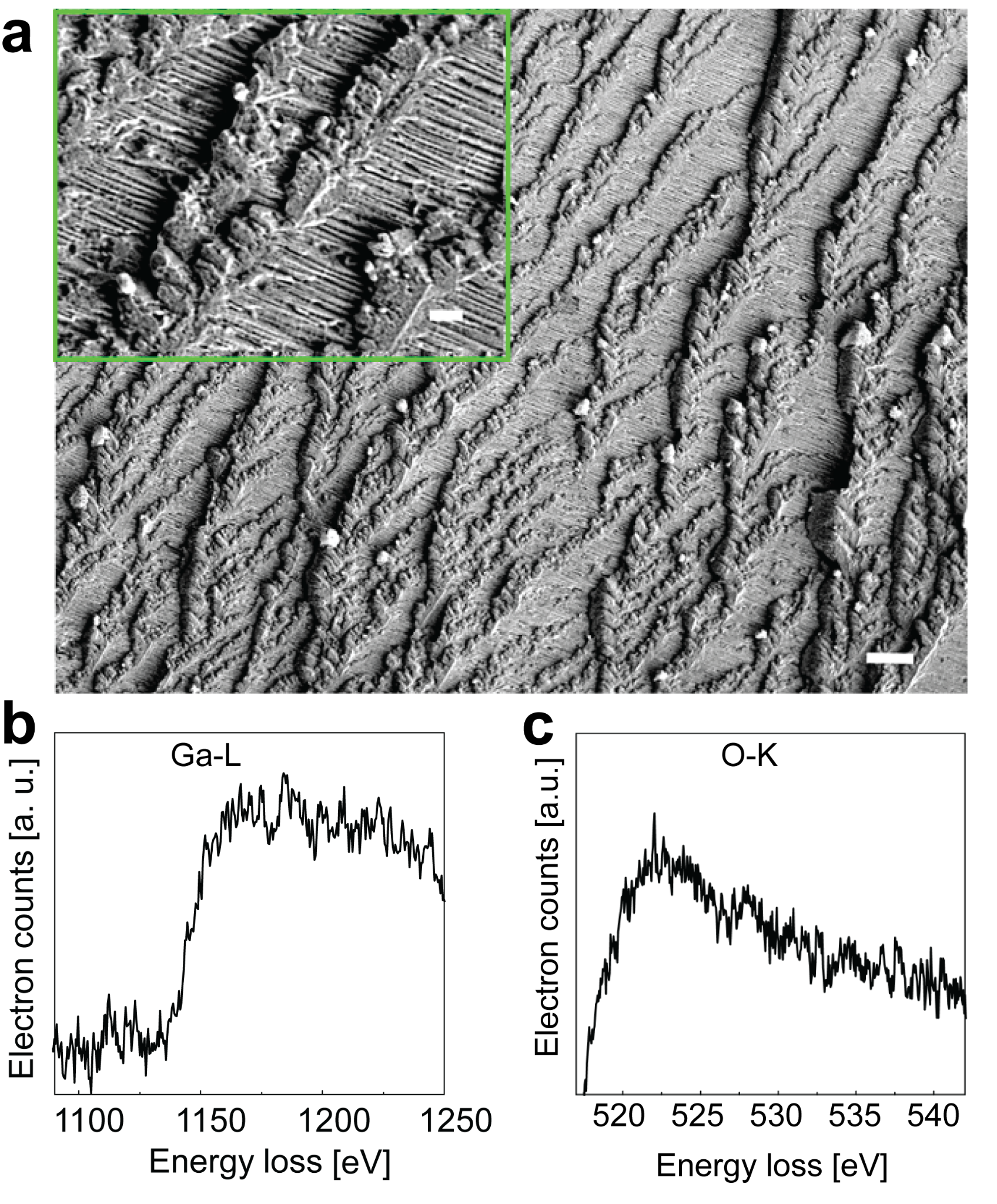


**Figure S3** | **Scanning electron microscopy and electron energy loss spectroscopy.** **a**, Scanning electron microscopy (SEM) of slowly cooled gallium droplets sandwiched between cleaned glass and PDMS substrate showing highly oriented superstructures of helical nanocrystals (filaments). Due to the strong supercooling effect, a seed crystal was employed to initiate the solidification of the droplet under confinement conditions. Scale bars are 2 µm and 300 nm (inset). **b,** Electron energy loss spectroscopy (EELS) spectrum recorded on the gallenene shown in Figure 1b. **c**, EELS spectrum obtained from the marked area on the gallenene crystal shown in Figure 1a.


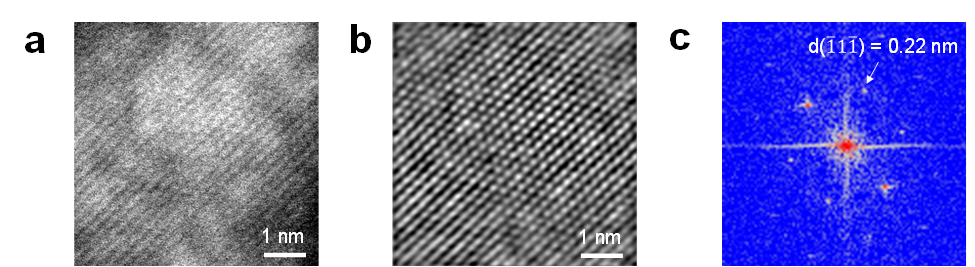


**Figure S4** | **High-angle annular dark field scattering. a-b**, Raw and double Gaussian filtered high-angle annular dark field (HAADF) scattering images of gallenene (a100). **c**, Fourier transform of gallenene (a100) shown in **a**. The d-spacing for ($\underline{1}1\underline{1}$) plane is measured to be 0.22 nm.

**
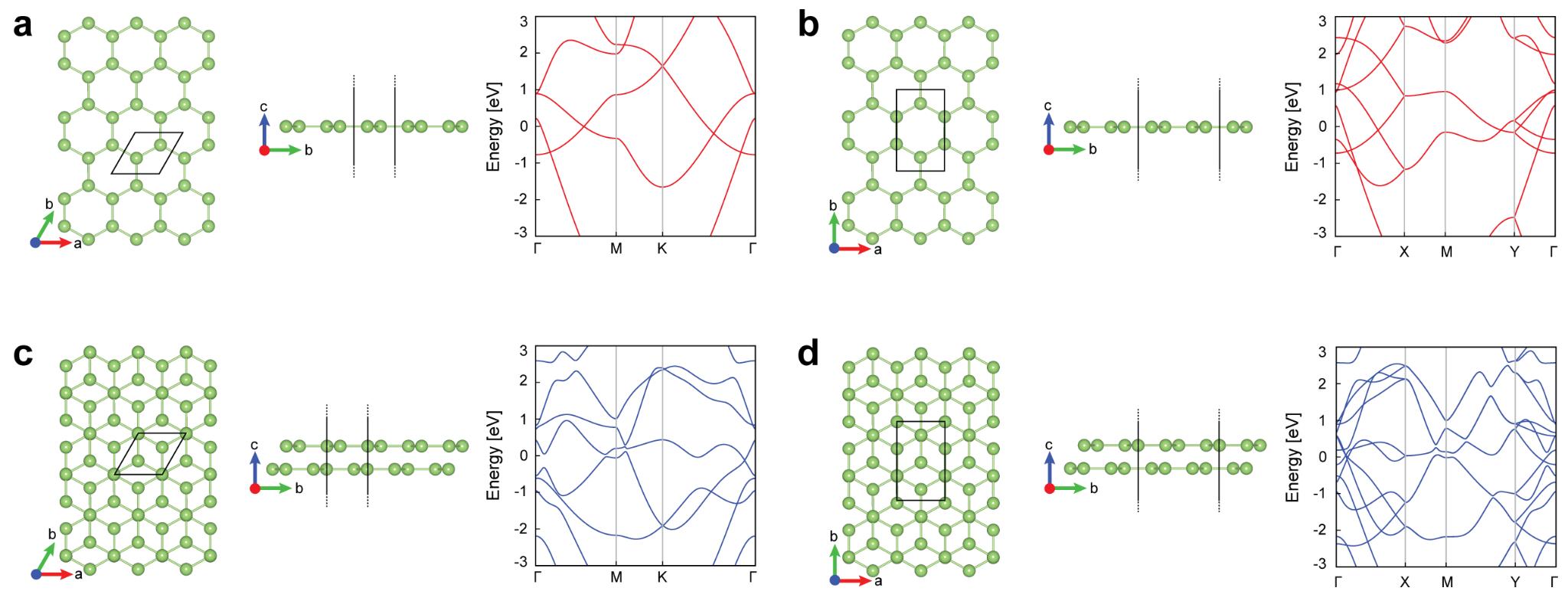
**

**Figure S5** | **Top and side views of the optimized crystal structures of a100 and their corresponding electronic band structures, shown from left to right**. **a**, Hexagonal and **b**, rectangular crystal structures of monolayer a100. **c**, hexagonal **d**, rectangular crystal structures of bilayer a100. The unit cells are outlined, respectively.


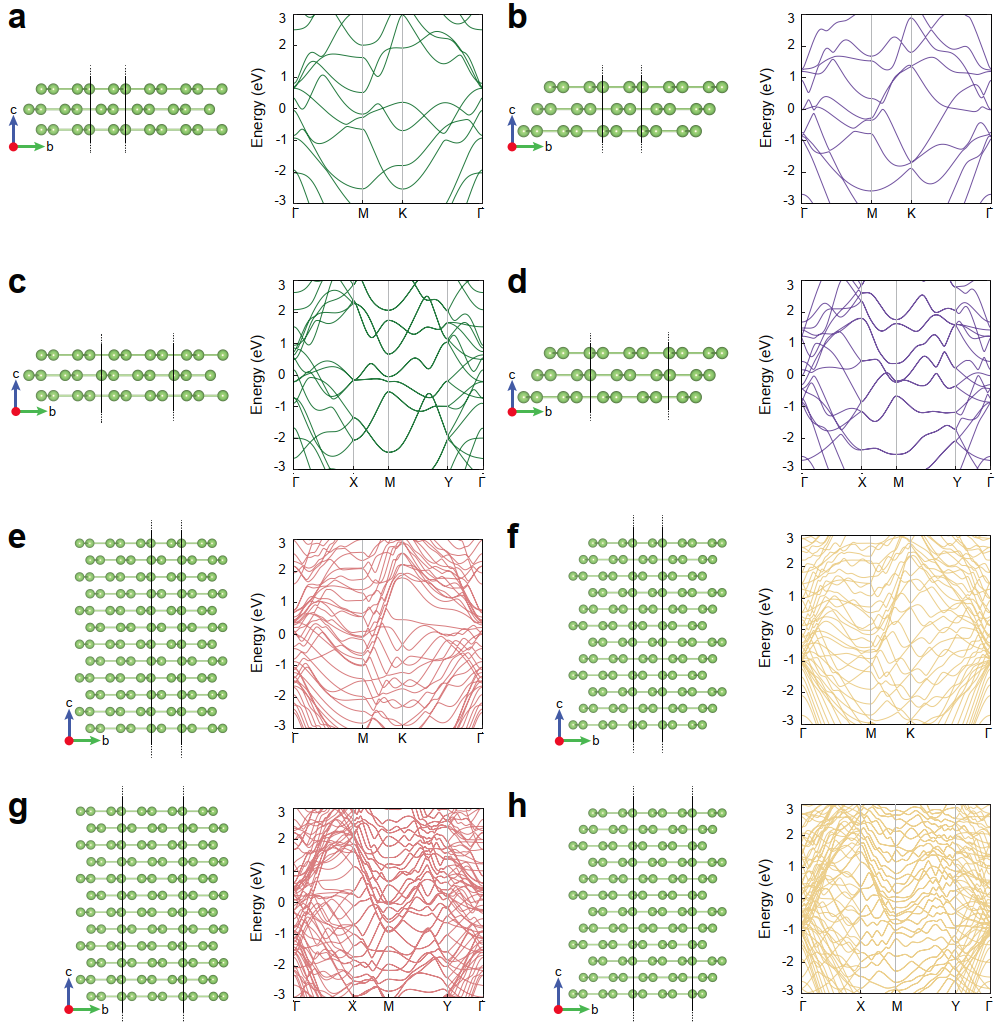


**Figure S6** | **Side views of the optimized trilayer and 12-layer a100 crystal structures and their corresponding electronic band structures**. **a-b**, **e-f**, Structures in the hexagonal unit cell. **c-d**, **g-h**, Structures in the rectangular unit cell. **a**, **c**, **e**, and **g** correspond to ABA stacking, while **b**, **d**, **f**, and **h** correspond to ABC stacking.

**
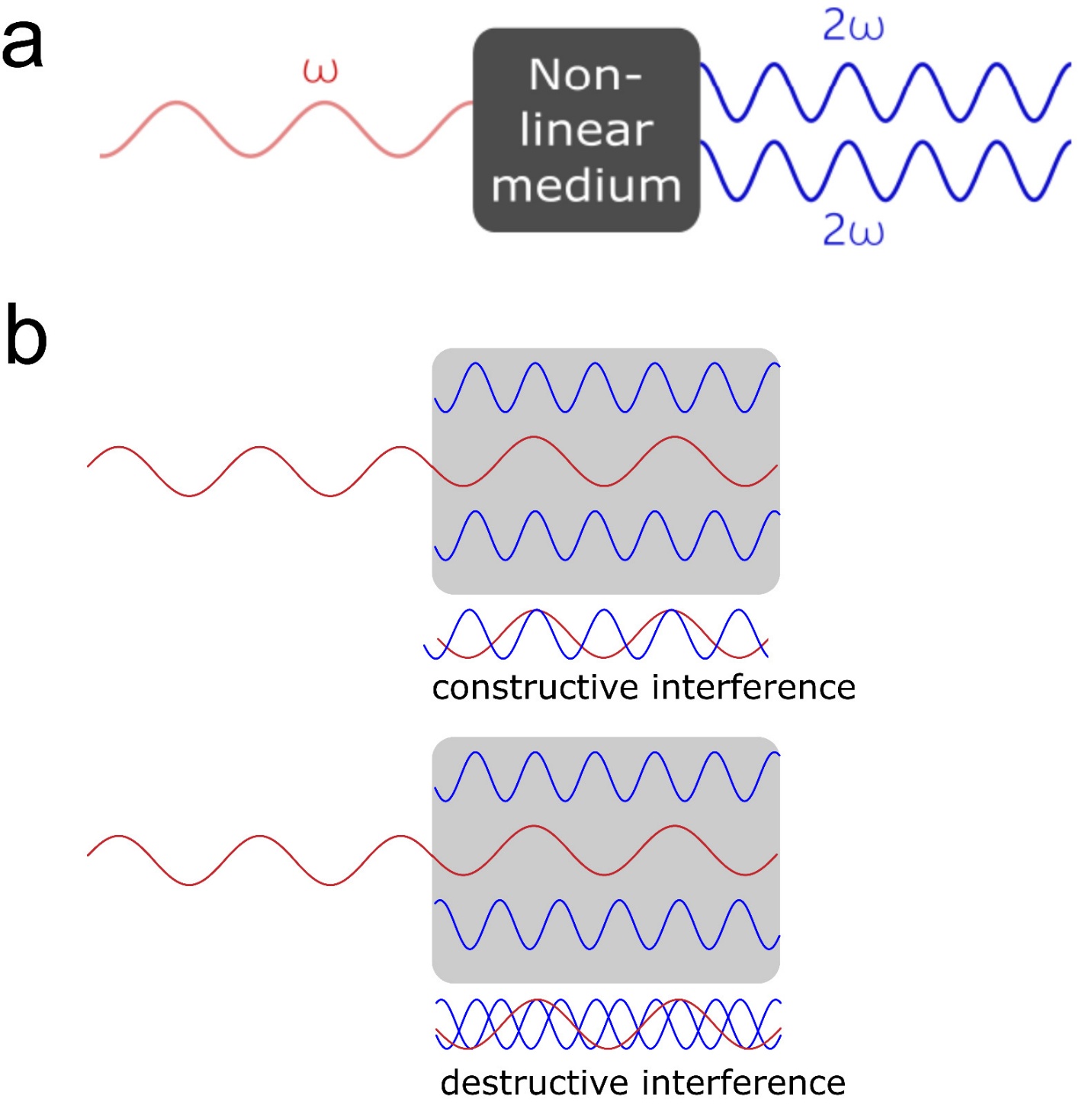
**

**Figure S7** | **Schematic of fundamental second harmonic generation mechanism. a**, Shows the harmonic signals generated after going through a nonlinear medium. **b**, Schematic showing the difference in signal generation when interacting with a constructive versus destructive interface for SHG. The phase velocity of fundamental wave and frequency doubled wave must be similar, in best case equal, for constructive interference to occur and SHG to exist in the far-field.


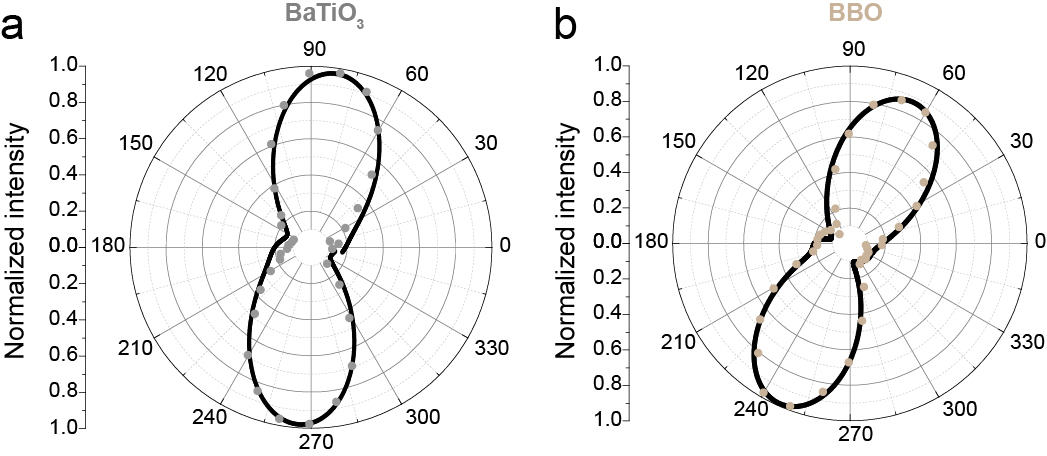


**Figure S8 | Rotational petals displayed for reference materials to demonstrate functionality of SHG microscope**. Second harmonic generation intensity response to changes in rotation for **a**, BaTiO3, and **b**, β-BaB2O4, respectively. SHG response peaks are shown in Figure 3d, e.

**
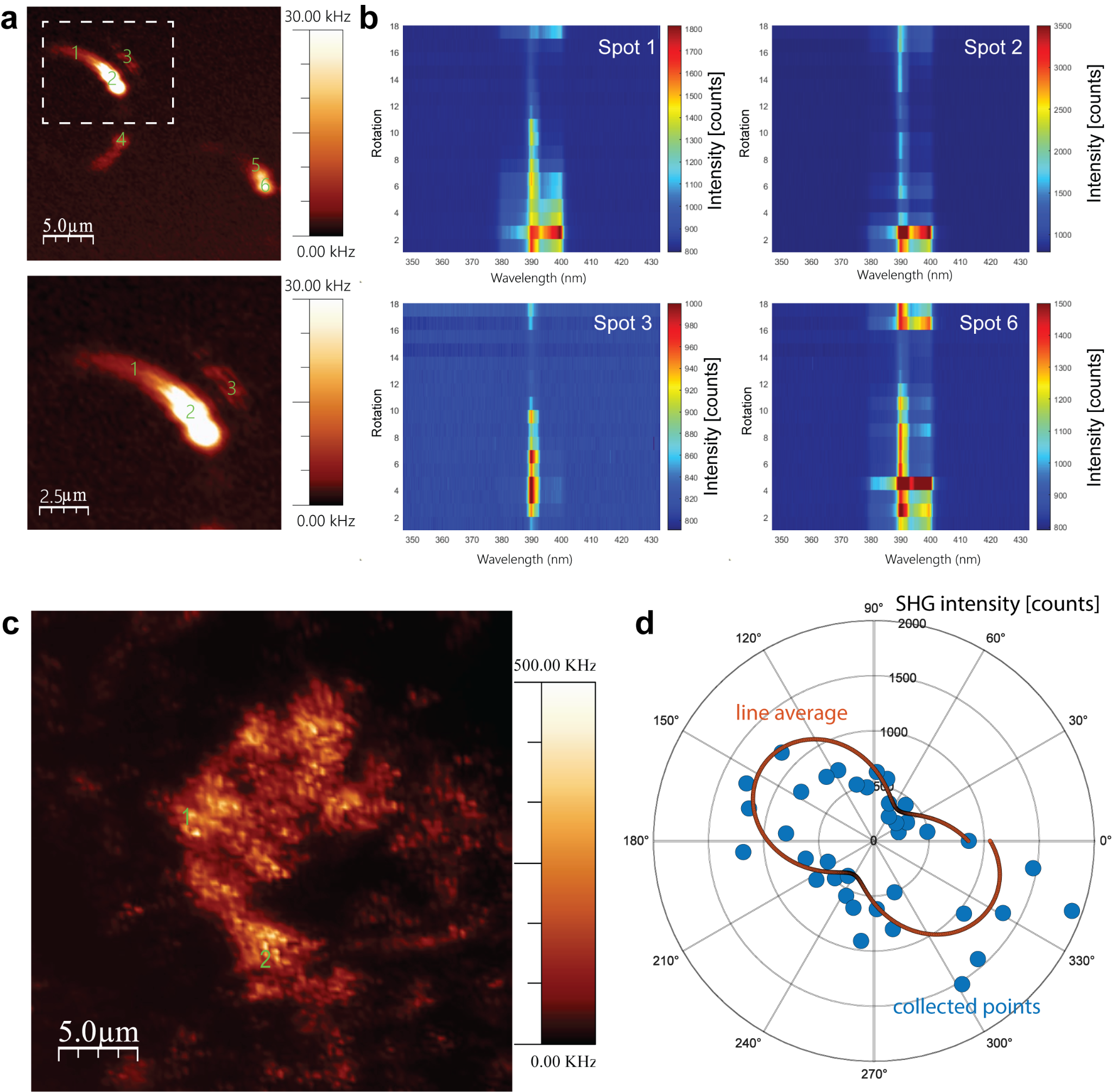
**

**Figure S9** | **Influence of polarization vector rotation of the linearly polarized beam. a**, Scan image of six different spots with a zoom in on spots 1-3 shown in the inset. **b**, Pseudocolor plots showing the SHG intensity for different beam electric field vector rotation angles. Rotation is given in steps of 10° on the y-axis. **c**, Scan image at 0° for an experiment with rotation from 0° to 360° in steps of 10° corresponding to the SHG shown in **d**. **d**, SHG intensity for beam electric field vector rotation from 0° to 360° in steps of 10°, averaged for both spots shown in **c**. The red line shows a nonlinear fit function.


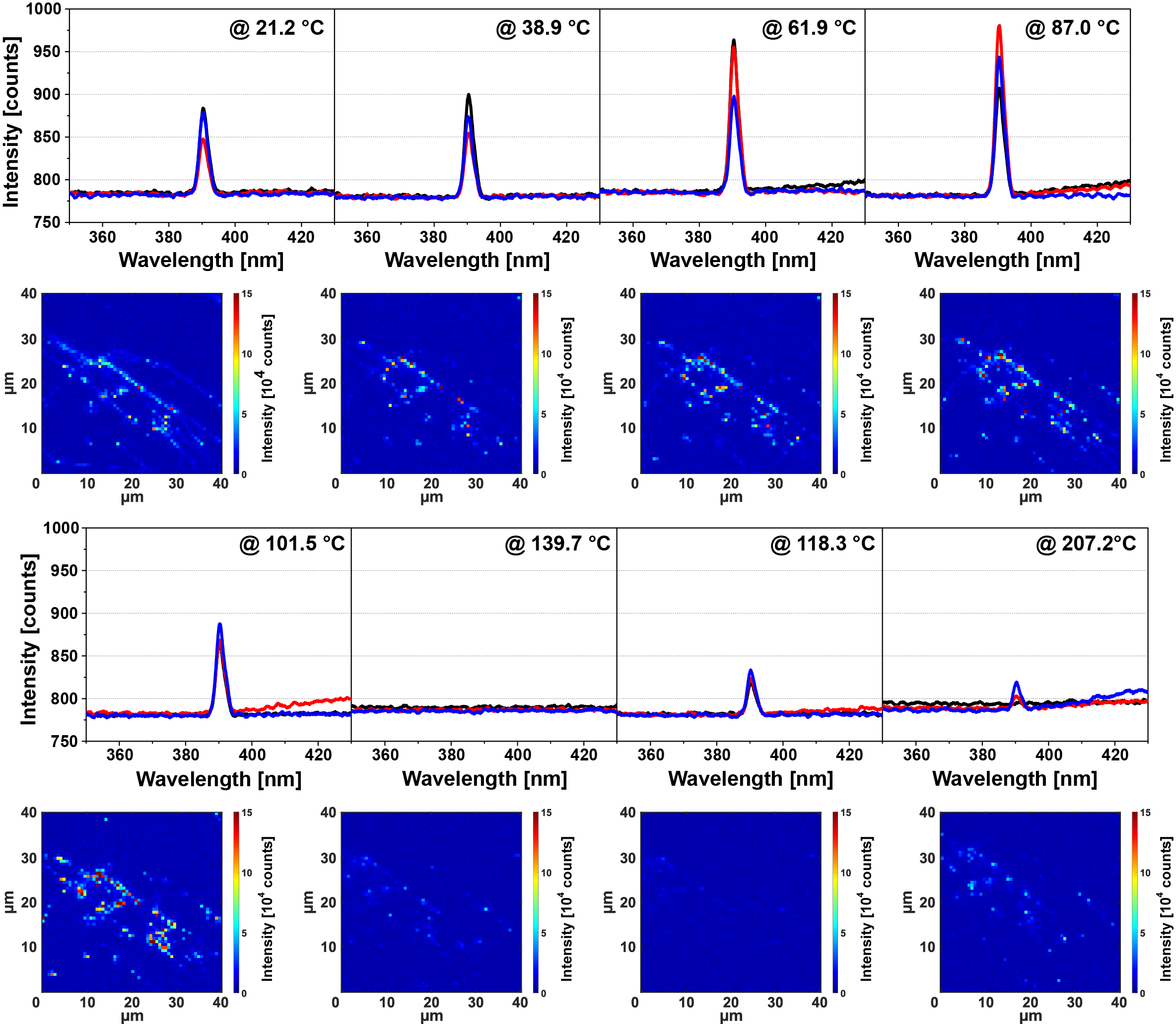


**Figure S10** | **Thermal perturbation and stability.** Sequence of nonlinear optical scan images and spectra containing SHG and photoluminescence information at different temperature values indicated in the legend of the SHG spectra. Temperature ranges from room temperature (top-left) to 207 °C (bottom right). Red, blue and black spectra correspond to one individual spot tracked over the whole range, respectively.

**
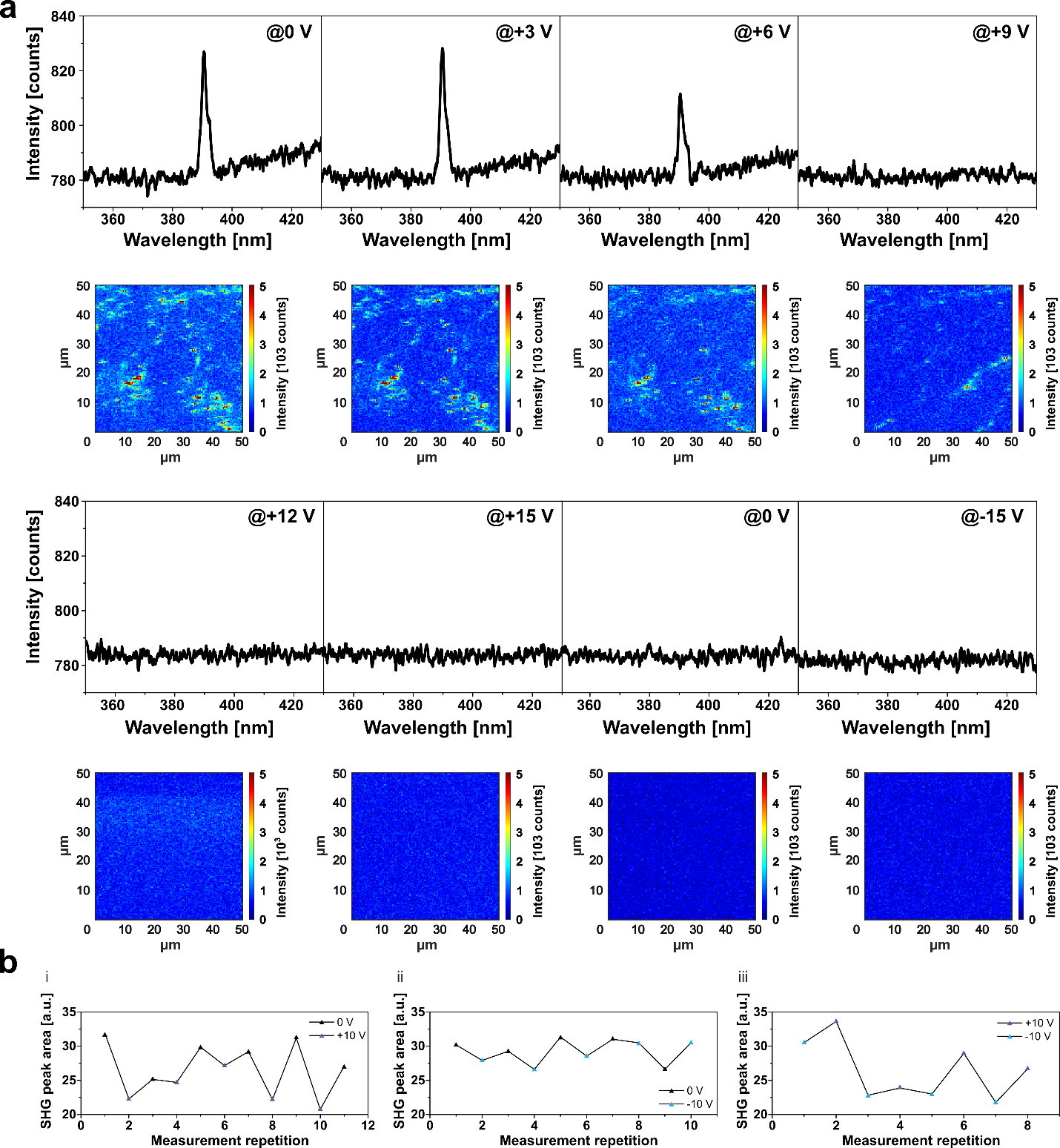
**

**Figure S11** | **Investigation of voltage sensitivity and stability.** **a**, Spectra and nonlinear optical scan images showing SHG and photoluminescence (PL) signals. Voltage applied to the SLG sandwich device is changed in steps of 3 V from 0 V to 15 V, then to -15 V. Voltages higher than 10 V, or -10 V in reference experiments, lead to a loss of gallium SHG; the signal is not recoverable once the threshold is exceeded. **b**, Average SHG response of all measured spots shown in Figure 6 (n = 10 (i)-(iii)) for (i) 0 V and +10 V, (ii) 0 V and -10 V, and (iii) +10 V and -10 V, including spots that show switching behavior and spots that do not.

**Figure S12** | **Sample preparation for PFM and working electrode for cyclic voltammetry.** The yellow circle represents the active area where dense SLG is deposited as a working electrode. The rest of the ITO surface is covered with Kapton tape to isolate the electrically conducting ITO glass from exposure to electrolyte. Wire connection is secured using conducting silver epoxy paste. The same sample preparation is employed for PFM measurement but without the Kapton tape layer on the ITO substrate to enable access to the conducting substrate.


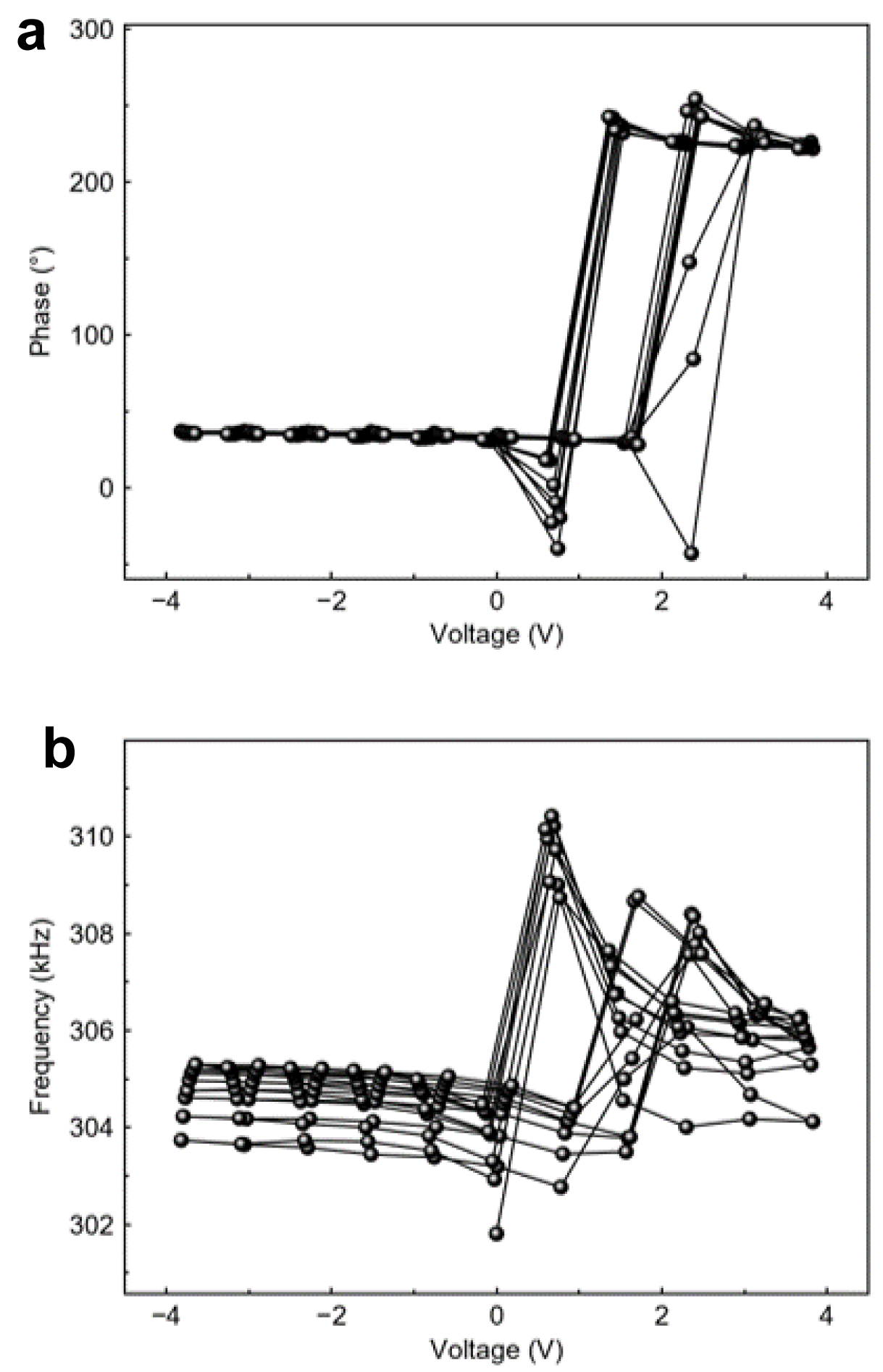


**Figure S13** | **PFM measurement of ferroelectric hysteresis.** ON-field phase loop and typical butterfly loop for the applied ±4 V dc bias. **a**, Amplitude loop, **b**, **c**, phase loop and d) frequency.

**Figure S14** | **Comparison of ferroelectric polarization switching profile.** The polarization switching curve of SLG. Overlay of current-voltage profile.

**Figure S15** | **Cyclic voltammetry (CV) and ferroelectric polarization profile obtained using 3-electrode configuration in PBS electrolyte recorded at 20 mV/s scan rate.**

**Figure S16** **| Cyclic voltammetry (CV) of pristine SLG indicating redox peaks of gallium at roughly -0.754 V vs Ag/AgCl. The scan rate was 20 mV/s.**


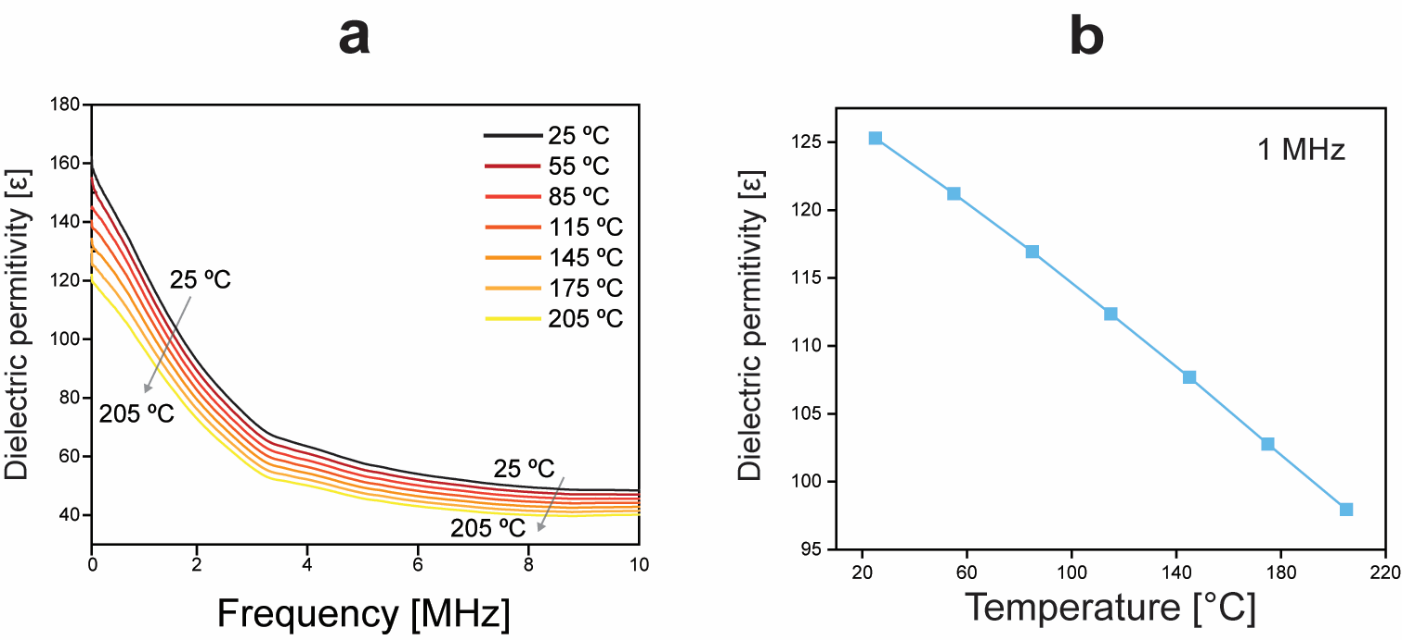


**Figure S17 | Dielectric characterization.** **a**, The dielectric permittivity measured across SLG samples with temperature variation from room temperature up to 205 ℃. The dielectric permittivity in S.I. units is F/m. **b**, Dielectric permittivity at 1 MHz obtained from e showing a linear behaviour.

**
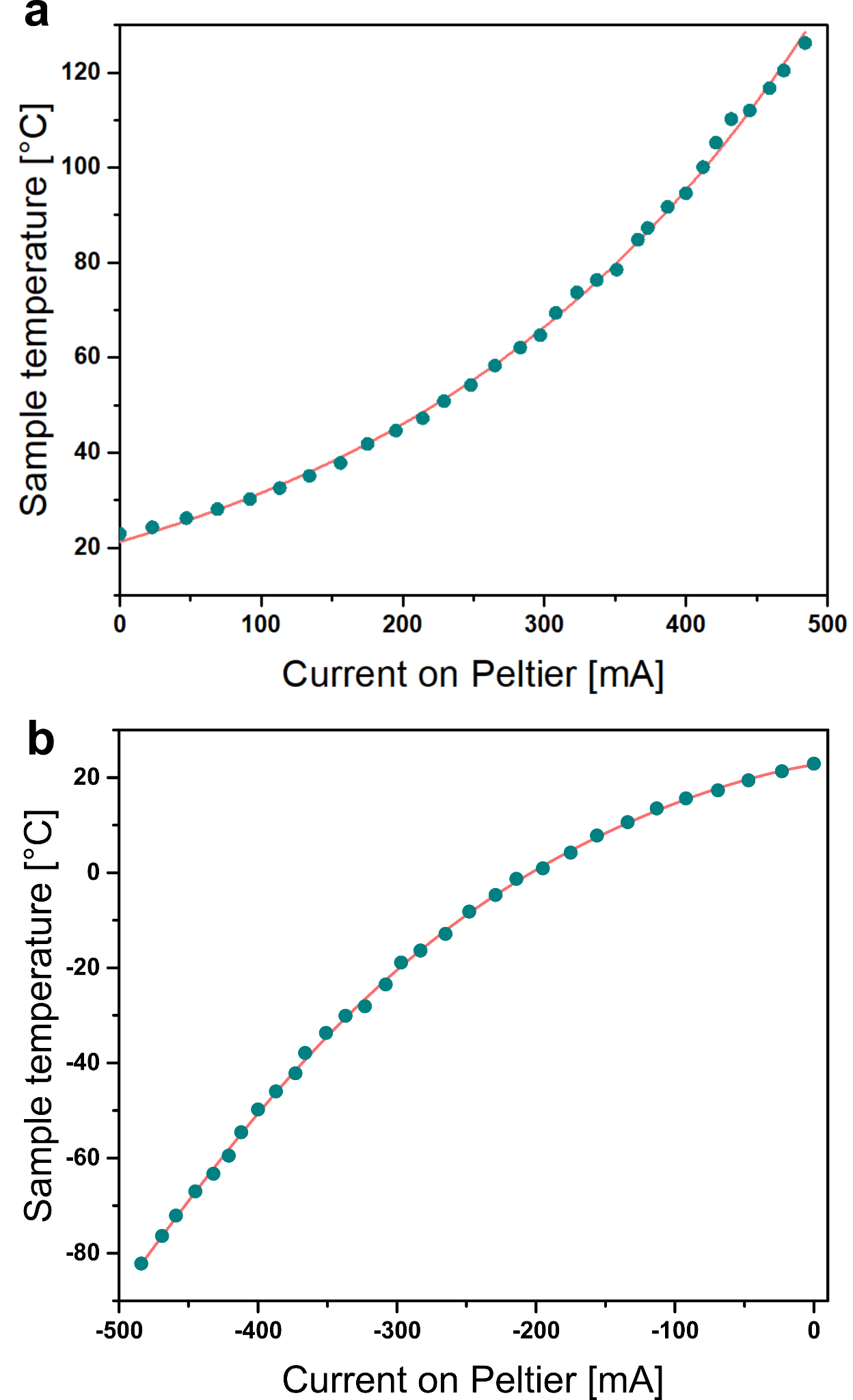
**

**Figure S18** | **Experimental heating and cooling curves of the SLG sandwich device mounted on a Peltier heating element. a**, The curve displays the current on Peltier in mA versus the sample temperature measured on top on the SLG sandwich device for the positive temperature range. **b**, The same as **a**, but with reversed polarity on the Peltier to cover the negative temperature range.


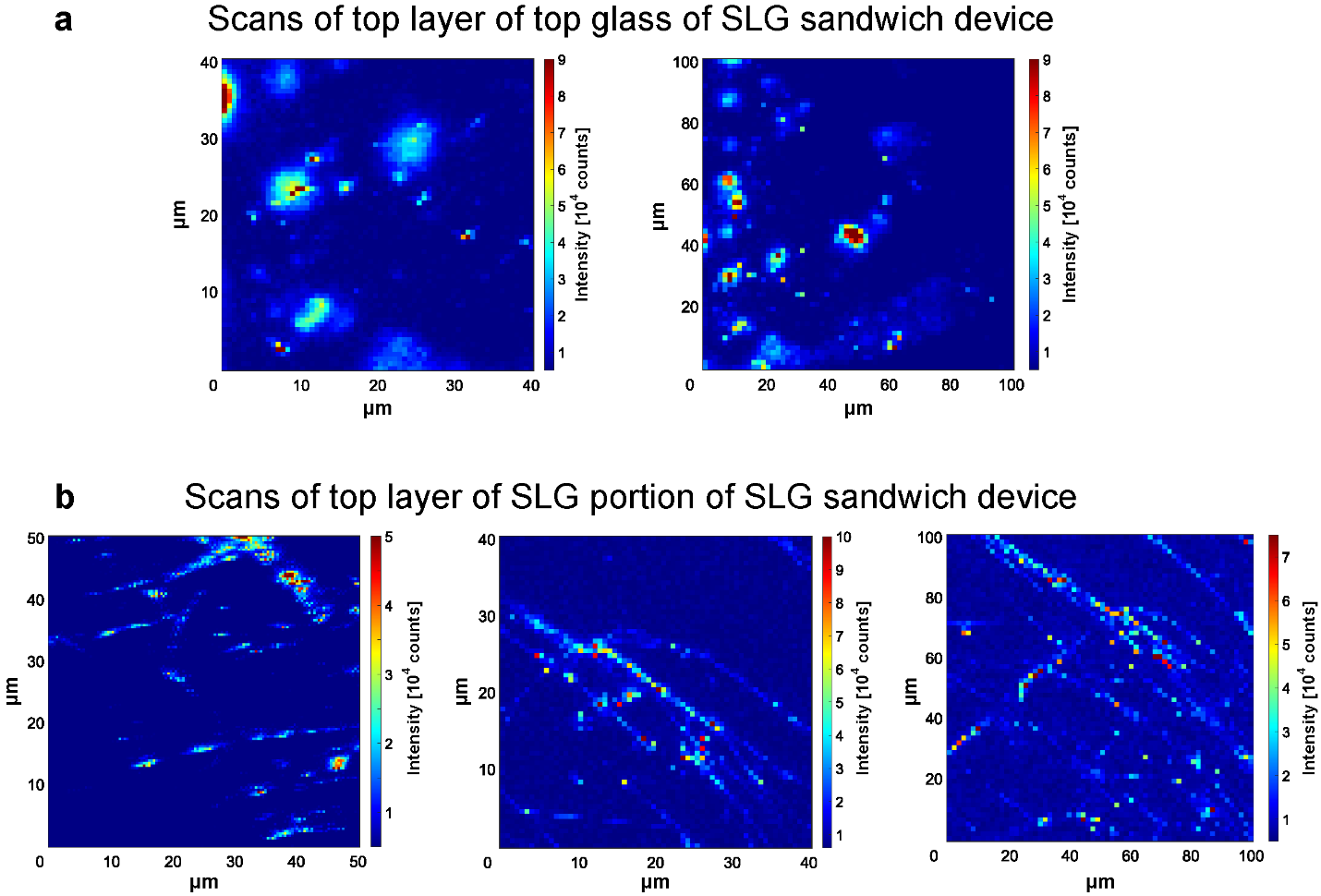


**Figure S19** | **Focal plane references.** **a**, APD scan images of the first focal plane located on the top layer of the SLG sandwich device. Bubbly structures are visible that are not affected by optical polarization, voltage or temperature changes. **b**, APD scan images of the second focal plane located at the phase boundary between liquid gallium and upper glass layer inside the SLG sandwich device. Strand-shaped structures can be observed that are sensitive to optical polarization, voltage or temperature changes.


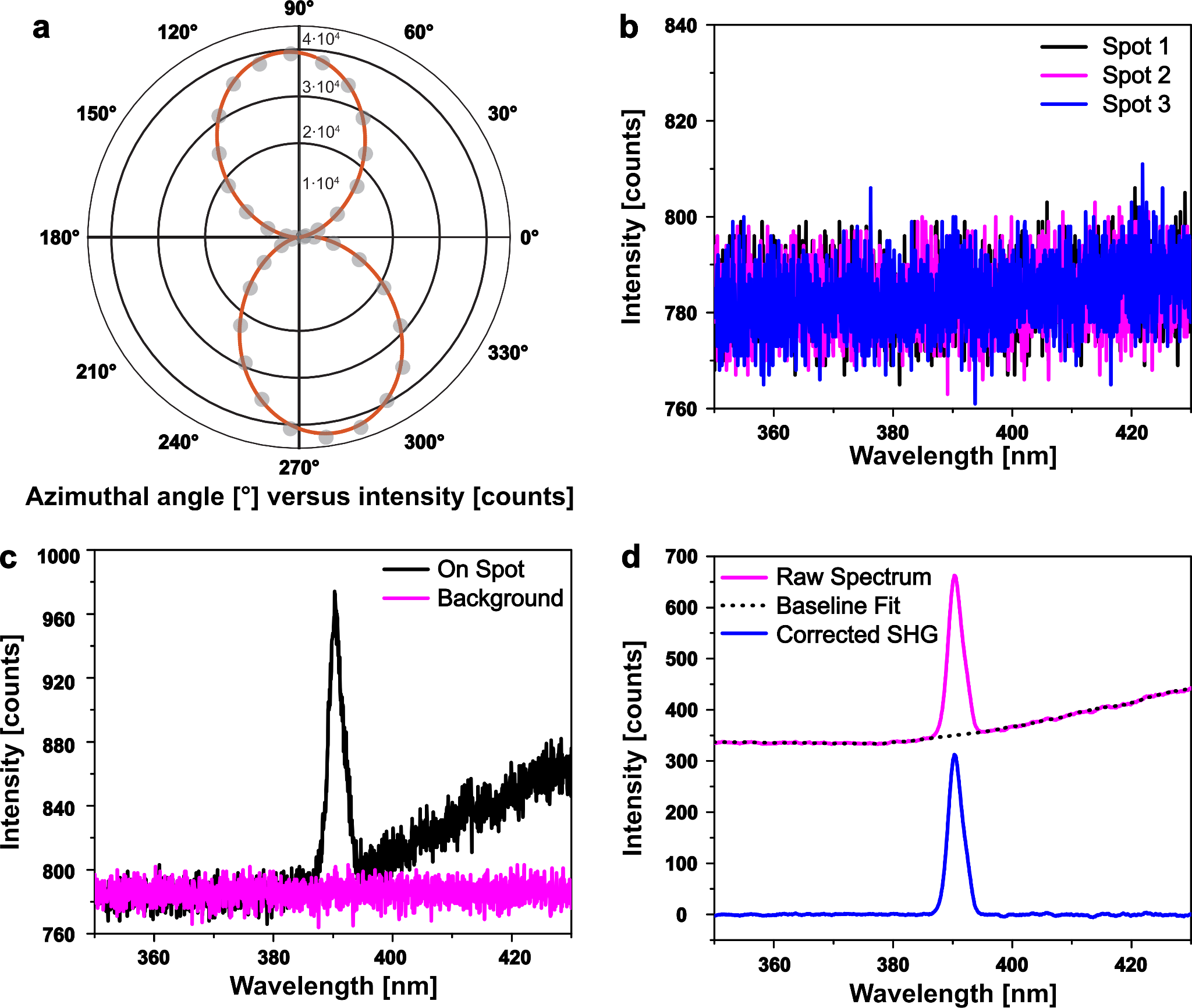


**Figure S20** | **References and SHG fitting with PL subtraction.** **a**, Polar plot of the linearly polarized laser scattering intensity measured with a Glan-Thompson analyzer at varying rotating angles. **b**, Plot showing the intensity in the SHG spectral range for the wrong focal plane shown in Figure S12a. **c**, Plot showing the intensity in the SHG spectral range taken on strand-shaped structures (On Spot’) visible in Figure S12b. The spectrum away from these structures is taken as the ‘Background’ for comparison. **d**, Plot showing the applied procedure for subtracting the photoluminescence-background from the pure SHG intensity.

**Supplementary Tables:**

**Table S1** | **Overview of sample properties used for different experiments.** Description of specific SLG sandwich devices used for various microscopy techniques.

| **Technique** | **Perturbation** | **Material** | **Substrate Thickness [µm]** | **Top Substrate Treatment** | **Bottom Substrate Treatment** | **Electrode** |
| --- | --- | --- | --- | --- | --- | --- |
| SHG | Angular | Glass | 120 | Coated, Unlined, Heat treated | Coated, Unlined, Heat treated | no |
| SHG | Voltage | Glass | 120 | Coated, Unlined, Heat treated | Coated, lined, Heat treated | yes |
| SHG | Thermal | Glass | 120 | Coated, Unlined, Heat treated | Coated, lined, Heat treated | no |
| TEM | N/A | Graphene | monolayer | N/A | N/A | no |
| Synchrotron | Voltage | Glass | 500 | Coated, Unlined, Heat treated | Coated, Unlined, Heat treated | yes |
| Synchrotron | Voltage | Glass | 500 | Coated, Unlined, Heat treated | Coated, Unlined, Heat treated | no |
| Synchrotron | Voltage | Glass | 500 | Coated, lined, Heat treated | Coated, lined, Heat treated | no |

**Supplementary Video:**

[**Video S1**](https://keeper.mpdl.mpg.de/f/f9069bbe14a14b519608/) | **Top-view of in-situ temperature change.** Videos are recorded on the sample stage in the self-built SHG microscope from top through the hole of the parabolic mirror, providing a clear view of the measured focal area. The scattering of the excitation infrared laser can be seen as red light. Scattering off many small spots is observed, which is stable for temperature changes up to 64 °C with slight mechanical vibrations of the camera. When the temperature increases to 87 °C, the area of interest begins to change visibly on the left side of the focal spot. Further temperature increases up to 207 °C lead to changes in the scattering behavior, which clearly proves structural changes at the sample surface, accompanied by the formation of liquid domains, which shows the phase change from M2 to M1. At 207 °C white light is switched on to observe the behavior during the steep temperature drop when the Peltier heating module is switched off. The sample gradually changes its phase back from M1 to M2.

# Supplementary References

[1] J. Fu, N. Yang, Y. Liu, Q. Liu, J. Du, Y. Fang, J. Wang, B. Gao, C. Xu, D. Zhang, A. J. Meixner, G. Gou, F. Huang, L. Zhen, Y. Li, *Advanced Functional Materials* **2024**, *34*, 2308207.

[2] E. Gürdal, A. Horneber, N. Shaqqura, A. J. Meixner, D. P. Kern, D. Zhang, M. Fleischer, *The Journal of Chemical Physics* **2020**, *152*, 104711.

[3] L. Pan, P. Miao, A. Horneber, A. J. Meixner, P.-M. Adam, D. Zhang, *Beilstein J. Nanotechnol.* **2022**, *13*, 572.

[4] L. Pan, P. Miao, A. Horneber, A. J. Meixner, P.-M. Adam, D. Zhang, *ACS Appl. Nano Mater.* **2023**, *6*, 6467.

[5] J. Wang, J. Butet, G. D. Bernasconi, A.-L. Baudrion, G. Lévêque, A. Horrer, A. Horneber, O. J. F. Martin, A. J. Meixner, M. Fleischer, P.-M. Adam, D. Zhang, *Nanoscale* **2019**, *11*, 23475.

[6] R. Wang, Q. Liu, S. Dai, C.-M. Liu, Y. Liu, Z.-Y. Sun, H. Li, C.-J. Zhang, H. Wang, C.-Y. Xu, W.-Z. Shao, A. J. Meixner, D. Zhang, Y. Li, L. Zhen, *Small* **2024**, *20*, 2305658.

[7] J.-P. Wang, Y.-Q. Fang, W. He, Q. Liu, J.-R. Fu, X.-Y. Li, Y. Liu, B. Gao, L. Zhen, C.-Y. Xu, F.-Q. Huang, A. J. Meixner, D. Zhang, Y. Li, *Advanced Optical Materials* **2023**, *11*, 2300031.

[8] J. Stadler, C. Stanciu, C. Stupperich, A. J. Meixner, *Opt. Lett., OL* **2008**, *33*, 681.

[9] J. Xiang, S. Li, Z. Sun, J. Chen, L. Chen, M. Pangmai, G. Li, S. Lan, *Advanced Optical Materials* **2021**, *9*, 2100675.

[10] A. Mooradian, *Phys. Rev. Lett.* **1969**, *22*, 185.

[11] E. Kim, A. Steinbrück, M. T. Buscaglia, V. Buscaglia, T. Pertsch, R. Grange, *ACS Nano* **2013**, *7*, 5343.

[12] M. Acosta, N. Novak, V. Rojas, S. Patel, R. Vaish, J. Koruza, G. A. Rossetti Jr., J. Rödel, *Applied Physics Reviews* **2017**, *4*, 041305.

[13] R. G. dos Santos, L. J. Q. Maia, C. B. de Araújo, L. de S. Menezes, *Chin. Opt. Lett., COL* **2018**, *16*, 041902.

[14] L.-Q. Yang, X.-M. Jiang, Y. Chen, B.-W. Liu, G.-C. Guo, *Advanced Optical Materials* **2024**, *12*, 2301897.

[15] G. Kresse, J. Furthmüller, *Phys. Rev. B* **1996**, *54*, 11169.

[16] J. P. Perdew, K. Burke, M. Ernzerhof, *Phys. Rev. Lett.* **1996**, *77*, 3865.

[17] P. E. Blöchl, *Phys. Rev. B* **1994**, *50*, 17953.

[18] H. J. Monkhorst, J. D. Pack, *Phys. Rev. B* **1976**, *13*, 5188.

[19] C.-K. Lee, L.-C. and Chien, *Ferroelectrics* **2000**, *243*, 231.
